# CO_2_ Capture and Low-Temperature Release by Poly(aminoethyl methacrylate) and Derivatives

**DOI:** 10.1021/acs.langmuir.1c02321

**Published:** 2021-12-09

**Authors:** Tony Tiainen, Jere K. Mannisto, Heikki Tenhu, Sami Hietala

**Affiliations:** Department of Chemistry, University of Helsinki, P.O. Box 55, Helsinki FIN-00014 HU, Finland

## Abstract

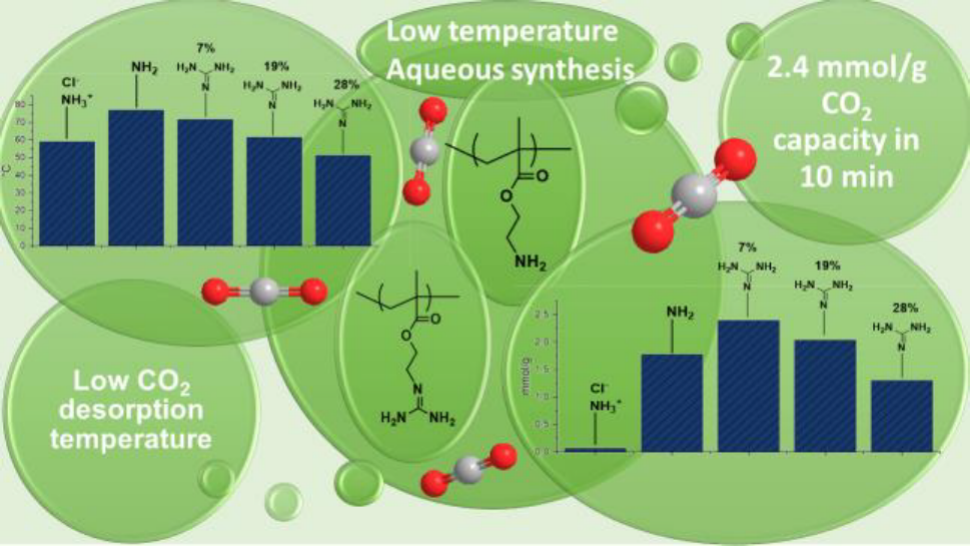

Poly(aminoethyl methacrylate) (PAEMA), poly(ethylene oxide)-*block*-(aminoethyl methacrylate) (PEO–PAEMA), and their guanidinylated derivates, poly(guanidine ethyl methacrylate) (PGEMA) and poly(ethylene oxide)-*block*-(guanidine ethyl methacrylate) (PEO–PGEMA), were prepared to study their capabilities for CO_2_ adsorption and release. The polymers of different forms or degree of guanidinylation were thoroughly characterized, and their interaction with CO_2_ was studied by NMR and calorimetry. The extent and kinetics of adsorption and desorption of N_2_ and CO_2_ were investigated by thermogravimetry under controlled gas atmospheres. The materials did not adsorb N_2_, whereas CO_2_ could be reversibly adsorbed at room temperature and released by an elevated temperature. The most promising polymer was PGEMA with a guanidinylation degree of 7% showing a CO_2_ adsorption capacity of 2.4 mmol/g at room temperature and a desorption temperature of 72 °C. The study also revealed relations between the polymer chemical composition and CO_2_ adsorption and release characteristics that are useful in future formulations for CO_2_ adsorbent polymer materials.

## Introduction

CO_2_ is a natural gas in the environment and is produced extensively by human activities, and it is considered one of the major contributing factors to the warming of the climate via the greenhouse effect.^1−5^ In addition, CO_2_ is used in various industrial processes and efficient capture and storage of CO_2_, and its use as a carbon source is a very important topic of research.^4,6−10^

Consequently, it is not surprising that recent times have seen a rise in interest regarding CO_2_ capture and separation.^10−25^ The studies explore various physical and chemical methods of adsorption of CO_2_ into solids^20,21,23,26−30^ or absorption into liquids.^31−33^ Liquid technologies are already well-established and applied in industry, yet it has been suggested that further development is needed.^24,34,35^ The main issues are the energy consumption of regeneration, running costs coming from evaporation, and the environmental impact of the solvents used.^10,32^ Solid adsorbents have the potential to be less energy demanding and more efficient compared to liquids.^24^ Solid monomeric, oligomeric, or polymeric adsorbents,^23,28,36,37^ polymer membranes,^26,29^ and hybrid systems^11,22,38,39^ are being developed to solve the issues with conventional liquid systems to allow lower regeneration temperatures, the avoidance of solvents, and an increase in the active surface area for more sustainable and efficient systems.

Among the polymeric materials, various nitrogen containing polymers have been investigated,^24^ especially polymers with amine functionalities.^40^ By using a polymer backbone with suitable side groups, CO_2_ adsorption kinetics, release temperatures, and material properties can be tailored. For example, Nie et al. prepared polyethyleneimide impregnated polyacrylamide composite beads for CO_2_ capture and managed to achieve a CO_2_ capacity of 2.64 mmol/g and a CO_2_ uptake of 90% in less than 10 min at temperatures between 50 and 125 °C.^22^ Goeppert et al. used polyethylenimine on fumed silica to achieve a 1.74 mmol/g capacity under similar humid conditions.^28^ Yue et al. prepared poly(*N*-isopropylacrylamide) microgel-based films having functional *N*-[3-(dimethylamino)-propyl] methacrylamide repeating units with large reversible CO_2_ capture from water saturated gas.^29^ In these examples, a high amount of amine groups incorporated on supports with suitable morphologies were used to achieve fast adsorption, high CO_2_ capacity, and low desorption temperatures. Kortunov et al. have shown that steric hindrance decreases the affinity of amine nitrogen to CO_2_, and thus, the morphology directly affects the adsorption and desorption performance.^41^ However, amine functionality is the key in interacting with the acidic CO_2_, especially if dry gas adsorption is preferable.^19,20,25,42,43^ Tertiary amines usually do not work well in dry adsorption, whereas secondary and primary amines are capable of forming ammonium carbamates with CO_2_ under anhydrous conditions increasing the dry adsorption performance with primary amines having a higher heat of adsorption and thermal stability.^25,35,44^ Another aspect to consider in CO_2_ adsorption is that a more basic group than amine could have stronger interactions with the acidic CO_2_. Superbases have been shown to interact with CO_2_ strongly.^18,45,46^ Guanidine, a type of superbase, has been used in an application to capture CO_2_ in solvated systems by Seipp et al.^31^ Guanidines have also been shown to increase CO_2_ uptake per mole in mixed base systems, where a nucleophilic amine is mixed with a non-nucleophilic Brønsted base, guanidine in this case.^18^ Overall, guanidine derivatives have been well-studied for CO_2_ capture as liquid mixtures.^47^ However, guanidines have not been studied extensively in dry and solid formulations.

Polyacrylates, such as poly(dimethylamino ethyl methacrylate) (PDMAEMA), are well-known for their versatility in materials applications as well as in gas separation membranes.^15,38,48−51^ However, PDMAEMA has a strong interaction with acidic moieties only in moist or wet conditions due to the nature of the tertiary amine.^35,44^ Poly(aminoethyl methacrylate) (PAEMA) is a similar polymer to PDMAEMA with the exception of having a primary amine functionality instead of tertiary one, and it has been studied for uses in biomedical research.^52−54^ As the primary amine of PAEMA allows a facile way to modify the side groups, we see it as a biologically safe, easily modifiable, and potent dry CO_2_ adsorbent.

By modifying the functional groups into guanidines, additional benefits such as an improved affinity toward CO_2_ and increased CO_2_ uptake per mole could be introduced to the material. Poly(ethylene oxide) (PEO) is a widely used biocompatible polymer that has been shown to enhance the gas separation and adsorption of CO_2_ in membrane applications.^8,12,55^

In this work, we prepared two polymers based on these hypotheses, PAEMA and PEO–PAEMA. Subsequent modifications of these polymers into poly(guanidine ethyl methacrylate) (PGEMA) and poly(ethylene oxide)-*block*-(guanidine ethyl methacrylate) (PEO–PGEMA) were made to adjust their affinity for CO_2_. PAEMA and PEO–PAEMA and the guanidine containing PGEMA and PEO–PGEMA were then characterized and tested for CO_2_ dry adsorption. The states of the side groups were taken into account by testing the polymers both as free bases and in salt form. The study shows that the dry CO_2_ capture of primary amine containing polymers can be improved by guanidinylation.

## Experimental Section

### Materials

Monomer 2-aminoethyl methacrylate hydrochloride (Sigma 90%, AEMA) was dissolved into acetate buffer (100 mg/mL) and stirred in an Al_2_O_3_/inhibitor remover mixture thoroughly. AEMA was then extracted by centrifuging (15 min, 4025 RCF) the solids out. Chain transfer agents (CTAs) 4-cyano-4-(phenylcarbonothioylthio)pentanoic acid (Sigma, CPA) and poly(ethylene oxide) methyl ether 2-(dodecylthiocarbonothioylthio)-2-methylpropionate (Sigma, 6000 g/mol, PEO-CTA) were used as received. All solvents were used as received except ultrapure deionized water, which was used fresh from filtration equipment (18 mΩ, UHQ H_2_O). Thermal initiator 2,2′-azobis[2-(2-imidazolin-2-yl)propane]dihydrochloride (Wako Chemicals, VA-044) was recrystallized from methanol with a small amount of UHQ H_2_O. 2-Ethyl-2-thiopseudourea hydrobromide (Sigma), sodium hydroxide (VWR aq. 0.1 M, NaOH), acetic acid (Merck EMSURE, CH_3_COOH), hydrochloric acid (Merck EMSURE fuming or WVR aq. 1 M, HCl), sodium carbonate (Merck EMSURE, NaCO_3_), sodium bicarbonate (Merck EMSURE, NaHCO_3_), and sodium acetate (Merck EMSURE, NaCH_3_COO) were used as received. Instrument grade CO_2_ (Linde, pure) and N_2_ (Linde, instrument 5.0, ≥99.999%) were used as received.

### Methods

#### Synthesis of Poly(aminoethyl methacrylate) (PAEMA) and Poly(ethylene oxide)-*block*-(aminoethyl methacrylate) (PEO–PAEMA)

**Scheme 1:**
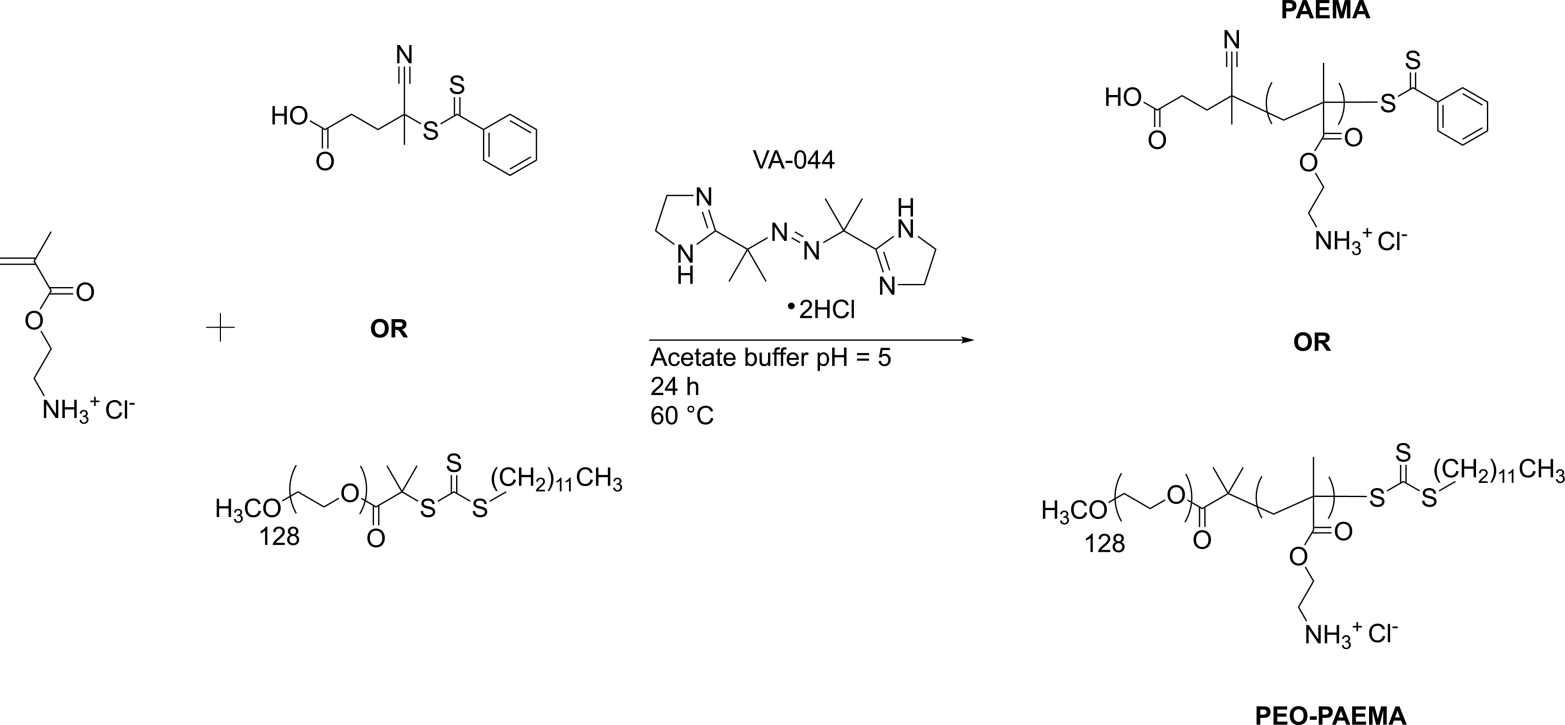
Synthesis of Polymers PAEMA and PEO–PAEMA

The synthesis of PAEMA and PEO–PAEMA (Scheme 1) was conducted with a modified version of a synthesis by Alidedeogly et al.^56^ (See Table S2.) Generally 14.1 mg of CPA (0.0503 mmol) or 300.9 mg of PEO-CTA (0.0503 mmol) and 4.1 mg of VA-044 (0.0126 mmol) were dissolved into 50 mL of AEMA dissolved in acetate buffer (30.190 mmol, 0.1 g/mL, Table S1) in a 50 mL round-bottom flask fitted with a septum. This solution was bubbled with argon under stirring for 1 h and immersed into a 60 °C oil bath to start the polymerization. The argon needle was lifted above the liquid surface 30 min after immersion and was completely removed after 15 min. The flask was left to stir for 24 h. The polymerization was quenched by opening the flask to air and immersing it into an ice bath for 15 min. The crude product was a mixed ammonium salt of acetate and chloride, which was converted to a chloride salt form by adding concentrated HCl and stirring until the reaction mixture was strongly acidic.

The acidic reaction solution was purified by dialysis in an MWCO 12–14 kDa cellulose membrane against aqueous 1 M HCl for 1 day and then against deionized water for 2 days and freeze-dried. ^1^H NMR: —C***H***_3_ (0.9–1.3 ppm), —C***H***_2_— (2 ppm), (—C***H***_2_—NH_2_) 3–3.6 ppm, PEO —C***H***_2_— (3.65 ppm), and O—C***H***_2_— (4.2 ppm).^57^^13^C NMR (Figure S8): —***C***H_3_ (17 ppm), —***C***H_2_—NH_2_ (38 ppm), —***C***—CH_3_ (44 ppm), —***C***H_2_— (53 ppm), O—***C***H_2_— (62 ppm), PEO —***C***H_2_— (69 ppm), and —***C***=O (178 ppm).

#### Regeneration of Free Base

**Scheme 2:**
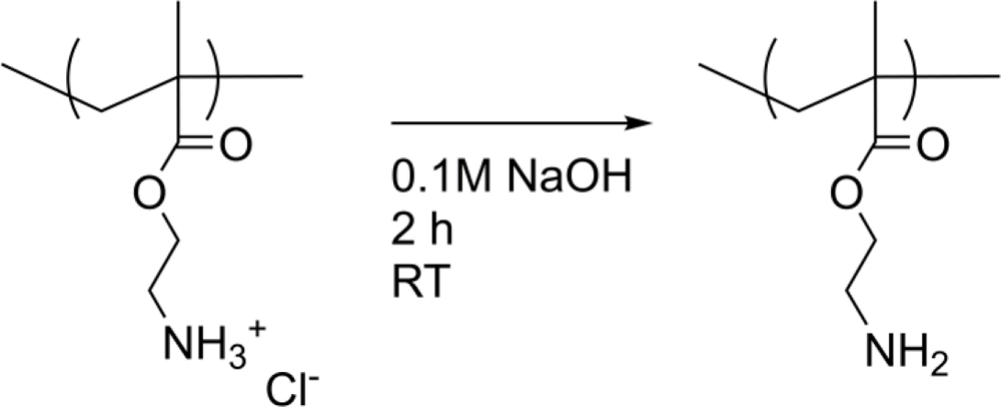
Regeneration of the Primary Amine Group

To convert the amine group from a less reactive salt into a free base, 500 mg of PAEMA or PEO–PAEMA was dissolved under stirring into 30 mL of 0.1 M NaOH for 2 h. (See Table S3.) The products were purified by dialysis against water in a 12 MWCO cellulose membrane and freeze-dried (Scheme 2). Regenerated polymers are denoted “REG” (Table 2).

#### Synthesis of PGEMA and PEO–PGEMA

**Scheme 3:**
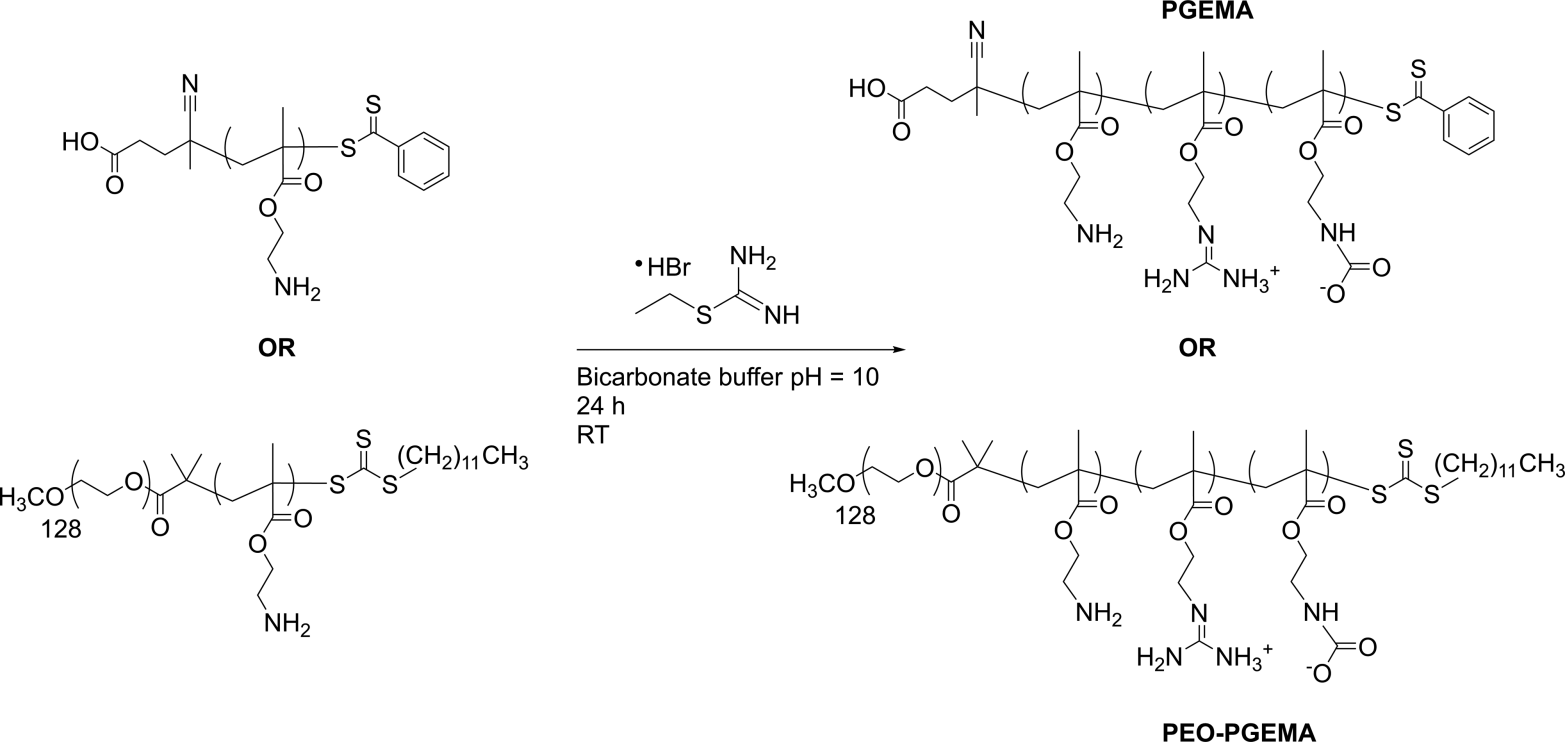
Synthesis of the Guanidinylated Polymers PGEMA and PEO–PGEMA

PGEMA (Scheme 3) and PEO–PGEMA (Scheme 3) were prepared using a modified version of a synthesis by Cheng et al.^54^ (See Table S4.) Generally, 200 mg (1.207 mmol) of PAEMA or PEO–PAEMA was dissolved into 10 mL of 0.1 M carbonate buffer. At the same time 0.2235 mg (1.207 mmol) of 2-ethyl-2-thiopseudourea hydrobromide was dissolved into 5 mL of buffer. Both solutions were combined and mixed at room temperature for 25 h. Conversion samples were taken at time intervals to determine the rate of reaction and degree of modification (Figures S1 and S2). The product was purified by dialysis against water in a 3.5 MWCO cellulose membrane and freeze-dried. Polymers with different amounts of guanidine were prepared by changing the reaction time and by using either the salt form or free base form of the polymer. ^1^H NMR: —C***H***_2_—N=C—N_2_H_4_ (3.6 ppm). ^13^C NMR: —N=***C***—(NH_2_)(NH_3_)^+^ (157 ppm).

### Characterization

FTIR spectra of dry powders were collected with a PerkinElmer Spectrum One instrument using an attenuated total reflection (ATR) setup. The measurement was made at room temperature taking at least four scans with the range 650–4000 cm^–1^.

NMR spectra were collected with either a Bruker Neo 400 or Bruker Neo 500 MHz spectrometer. NMR samples were prepared in deuterated water (D_2_O) at a 10–50 mg/mL concentration. Samples for measurements under a CO_2_ atmosphere were prepared by first flushing them in a Schlenk line under CO_2_ for 10 min and leaving them to stir for 1 h to saturate. Saturated samples were transferred to sealable Young’s NMR tubes and measured. After the measurements, the tubes were opened to air and placed into a preheated 60 °C oil bath for 1 h. After cooling, the samples were measured again. The molar mass for PEO–PAEMA was determined by comparing the known PEO signal at 3.7 ppm to PAEMA group signals.

Asymmetric flow field flow fractionation (AF4) was measured with a Wyatt Eclipse AF4 system running aqueous 50 mM NaNO_3_ + 0.05 mM NaN_3_ through the frit inlet (FI) channel and a 10 kDa MWCO regenerated cellulose membrane. Wyatt DAWN HELEOS II MALS and Wyatt REX RI detectors were used to obtain raw data. Raw data was processed with Astra version 6.1.7. Samples were prepared in the eluent 1 day before the measurement with a concentration of 1 mg/mL. Filtration of the sample was conducted just before the measurement using 0.45 μm PVDF filters.

The refractive index increment, d*n*/d*c*, was measured from a series of concentrations of PAEMA in running eluent using the Wyatt REX RI detector and was determined to be 0.6841 g/mL. The running parameters and fractograms are shown in the Supporting Information (Figure S3 and Table S5).

Thermogravimetric (TG) measurements were made with a Netzsch STA 449 F3 Jupiter instrument under a nitrogen or carbon dioxide atmosphere. The samples (5–8 mg) were placed in Al_2_O_3_ crucibles and equilibrated by three vacuum/fill cycles at 25 °C. The samples were heated without gas flow from 30 to 100 °C and cooled back to 30 °C at 10 °C/min with 10 min isothermals at both 30 and 100 °C. During cooling, the chamber was purged for 15 min with 250 mL/min N_2_ or 121 mL/min CO_2_. This cycle was repeated three times. A 20 mL/min protective N_2_ flow was constant during measurements. Onsets and adsorption/desorption kinetics were determined by a linear fit of the data and a determination of intersects and slopes (Figures S11–S14). Polymer degradation studies were made from 30 to 500 °C at 10 °C/min under N_2_.

Differential scanning calorimetry (DSC) measurements to determine the heat of desorption were made with a TA Instruments DSC Q2000 instrument. Samples were saturated with CO_2_ and prepared in hermetic aluminum crucibles directly from TGA measurements. The samples were equilibrated at −65 °C and heated from −65 to 180 °C. The heat of desorption was determined by fitting an integral starting from 50 °C and ending at 180 °C corresponding to the desorption temperature in Universal Analysis software from TA Instruments (Figures S15 and S16).

The pH values of the buffer solutions were measured at room temperature with a VWR Phenomenal IS2100L instrument using WTW or WVR electrodes calibrated within 1 day of the measurement with pH 4, 7, and 10 buffers from VWR.

## Results and Discussion

### Polymer Synthesis and Characterization

Polymer syntheses and subsequent modifications are shown in Tables 1 and 2 and Tables S2 and S4. The polymerization conditions were chosen so that the amino functionalities in the AEMA repeating units are in hydrochloride salt form during the reaction (Scheme 1). This is because the less reactive nature of the salt form compared to the free base helps to avoid aminolysis of the chain transfer agents.^58^ Fractograms (Figure S3) show the monomodal profile, and NMR spectra (Figures 2 and 3 and Figures S6 and S7) confirm the structures. To convert the amine hydrochlorides back to the free base form, parts of the samples were regenerated in aqueous 0.1 M NaOH and studied alongside of the rest of the samples (Scheme 2). When comparing the main and side chain ^1^H NMR signal integrals, no signs of hydrolysis can be observed. The amine content determined by titration of regenerated samples correlates well with the theoretical amount of amines, indicating successful regeneration (Table S6).

**Table 1:** Synthesis of Polymers

polymer	PAEMA	PEO–PAEMA
M:CTA:init.	600:1:0.25	600:1:0.25
reaction time (h)	24	24
conversion (%)	72	76
*M*_n_ (g/mol)	71 750 (theoretical), 23 400 (AF4), 117 700^a^ (NMR)	81 500 (theoretical), 66 500 (AF4), 76 100 (NMR)
PDI	1.4	3.0

a A limited end-group solubility overestimates the molar mass in NMR.

**Table 2:** Polymer Characteristics: Regenerated Polymers Are Labelled REG, and Modified Polymers Are Marked as PGEMA for Homopolymer and PEO–PGEMA for Block Copolymer Followed with the Modification Degree

polymer label	modification degree^a^ (%)	reaction time (h)
PAEMA	0	24
PAEMA REG	0	2
PGEMA 7^b^	7	6
PGEMA 19^b^	19	24
PGEMA 28^c^	28	25
PEO–PAEMA	0	24
PEO–PAEMA REG	0	2
PEO–PGEMA 11^b^	11	6
PEO–PGEMA 22^b^	22	25
PEO–PGEMA 29^c^	29	25

a The modification degrees were calculated from ^1^H NMR spectra by comparing the shifted CH_2_ proton signal at 3.6 ppm to the total of amount of protons next to the functional group (3, 3.4, and 3.6 ppm).

b Salt form polymer was used in modification.

c Free base polymer was used in modification.

The modification with guanidine progresses for both the salt and regenerated form of the polymers. This is due to the basic environment during the guanidinylation reaction that regenerates most of the primary amine side groups in the salt form. The conversion from amine to guanidine was monitored during the reaction, and the amount of guanidine groups was determined from NMR (Figures S1 and S2). This revealed that using the free base polymer as the starting reagent yields higher modification degrees with shorter reaction times.

This is most probably due to the readily available amine in the free base polymer compared to the polymer salt that regenerates first before reacting further. Further, the synthesis of PGEMA from PAEMA for over 24 h yielded under 20% guanidinylation, but PGEMA from PAEMA REG in the same time frame yields over 25% guanidinylation. With PEO–PGEMA, the trend is the same, 20% with PEO–PAEMA and almost 30% with PEO–PAEMA REG (Figure S2). Compared to an earlier report^54^ with the highest degree of guanidinylation around 46%, our values are lower. However, the previous authors determined the guanidinylation degrees via a trinitrobenzenesulfonic acid assay which is not directly comparable to NMR. This knowledge of kinetics could prove useful when a certain modification degree is desired.

**Figure 1:**
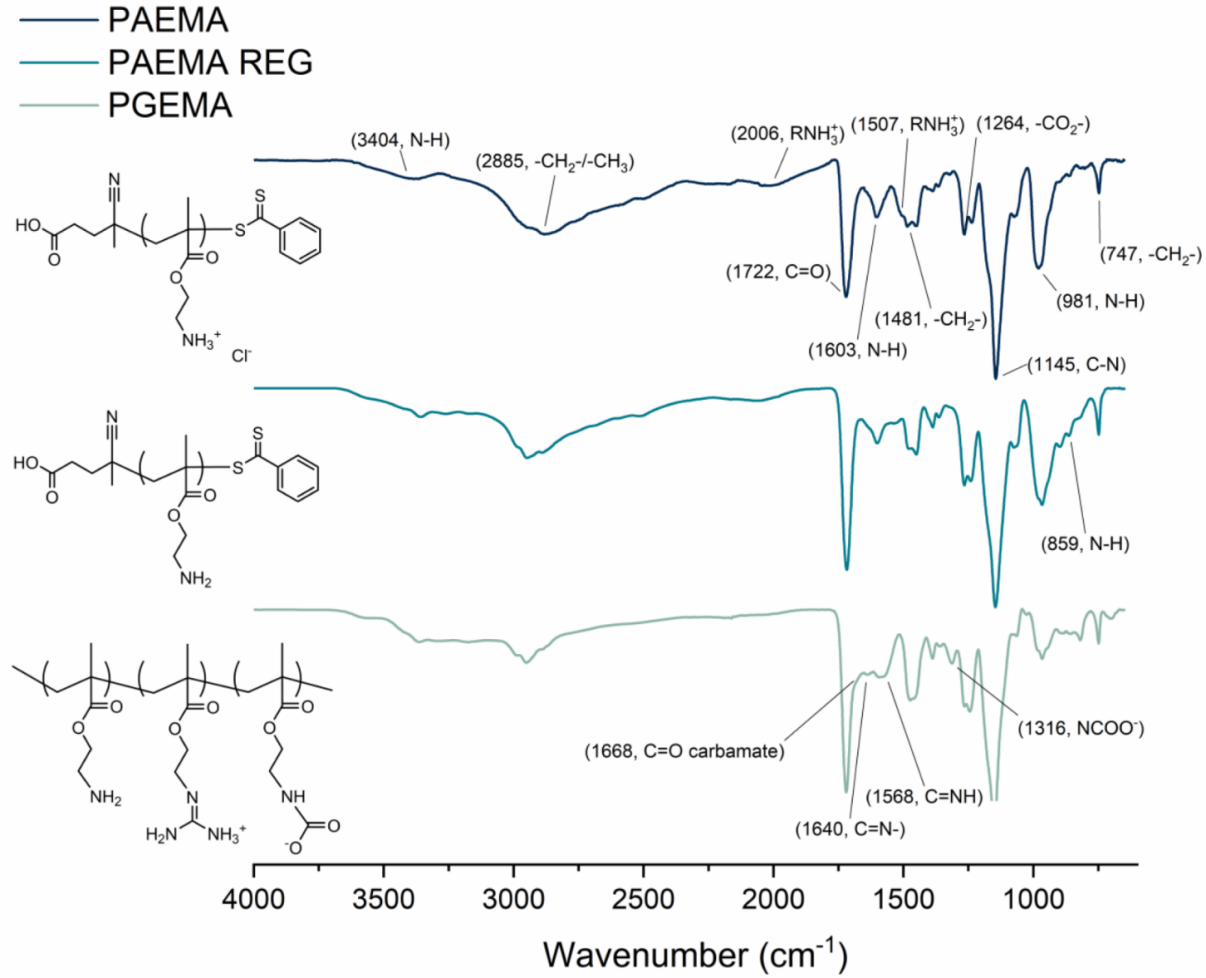
IR spectra of PAEMA, PAEMA REG, and PGEMA.

The IR of PAEMA reveals absorptions of the acrylate and amine parts of the polymer (Figure 1). More precisely, the most significant signals of acrylates can be seen at 2885 cm^–1^ (—CH_2_—), 1722 cm^–1^ (C=O), and 1481 cm^–1^ (—CH_2_—).^59^ A disappearance of the absorptions of PAEMA REG at 2006 and 1507 cm^–1^ (RNH_3_^+^) after regeneration confirms the successful removal of most salts (Figure S4). In PGEMA, these signals do not reappear, which suggests that the amino groups in the modified polymer are mostly in the free base state. An appearance of absorptions at 1568 (C=NH) and 1640 cm^–1^ (C=N—) confirms the presence of guanidine side groups. Also, absorptions at 1674 (C=O) and 1314 cm^–1^ (NCOO^–^) indicate the presence of carbamates.^42^ Similar results are seen in the cases of PEO–PAEMA, PEO–PGEMA REG, and PEO–PGEMA with the exception of the ether signals from the PEO chain at around 1000 cm^–1^ (Figure S5).

**Figure 2:**
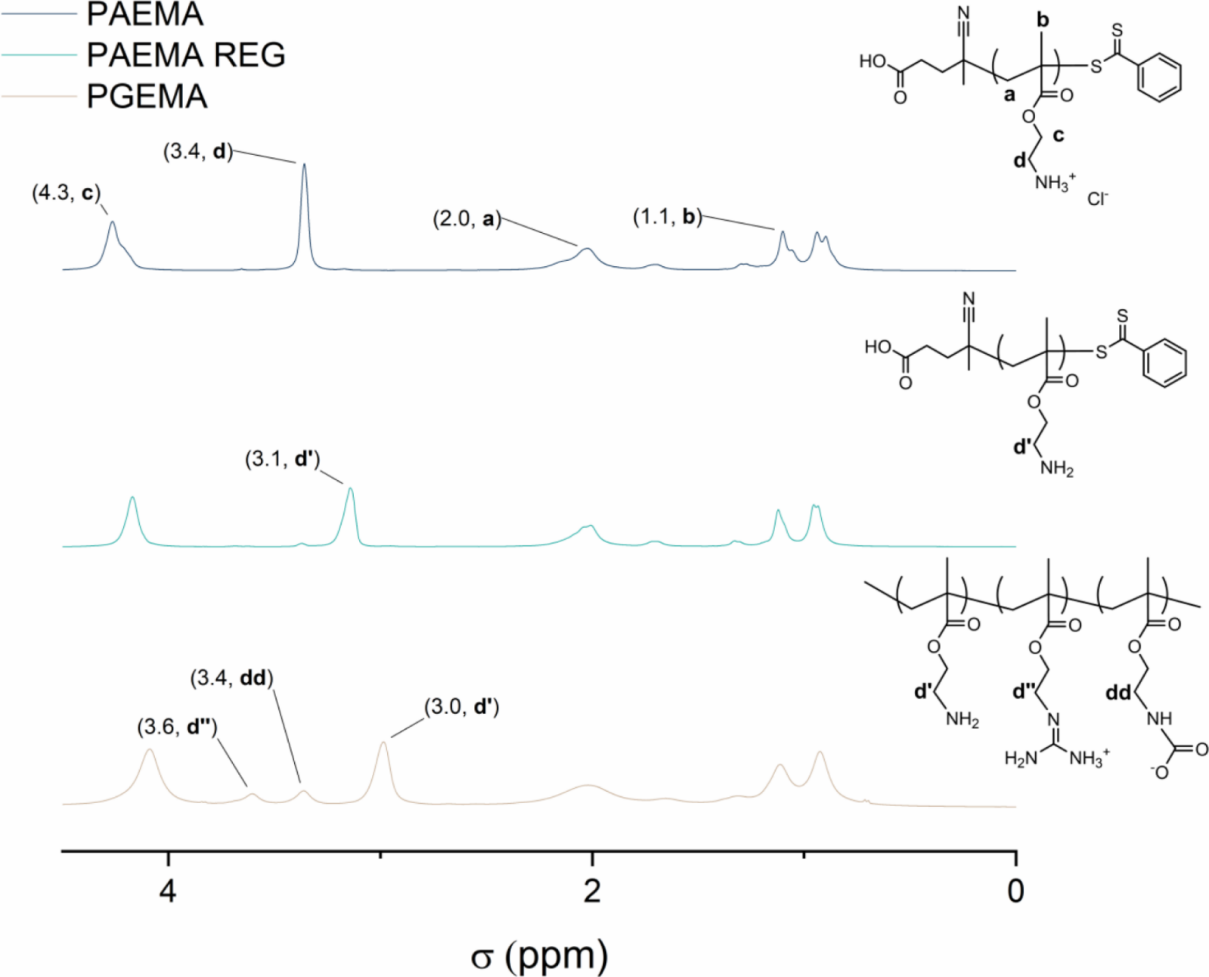
^1^H NMR spectra of PAEMA, PAEMA REG, and PGEMA.

The ^1^H NMR spectra are in line with the IR data (Figure 2). Notably, the chemical shifts from the protons next to the amine at 3–3.6 ppm (—C***H***_2_—NH_2_) are significantly affected depending on the state of the amine. In PAEMA, the CH_2_ signal next to the amine at 3.4 ppm shifts to 3.1 ppm when regenerated into PAEMA REG (Figure 2). In PGEMA, we see three signals for these protons, one being the signal from guanidinylated side groups at 3.6 ppm (—C***H***_2_—N=C—N_2_H_4_), carbamate at 3.4 ppm (—C***H***_2_—NH—COO^–^), and amine at 3.0 ppm (—C***H***_2_—NH_2_), revealing the mixed side group nature of the modified polymer.

**Figure 3:**
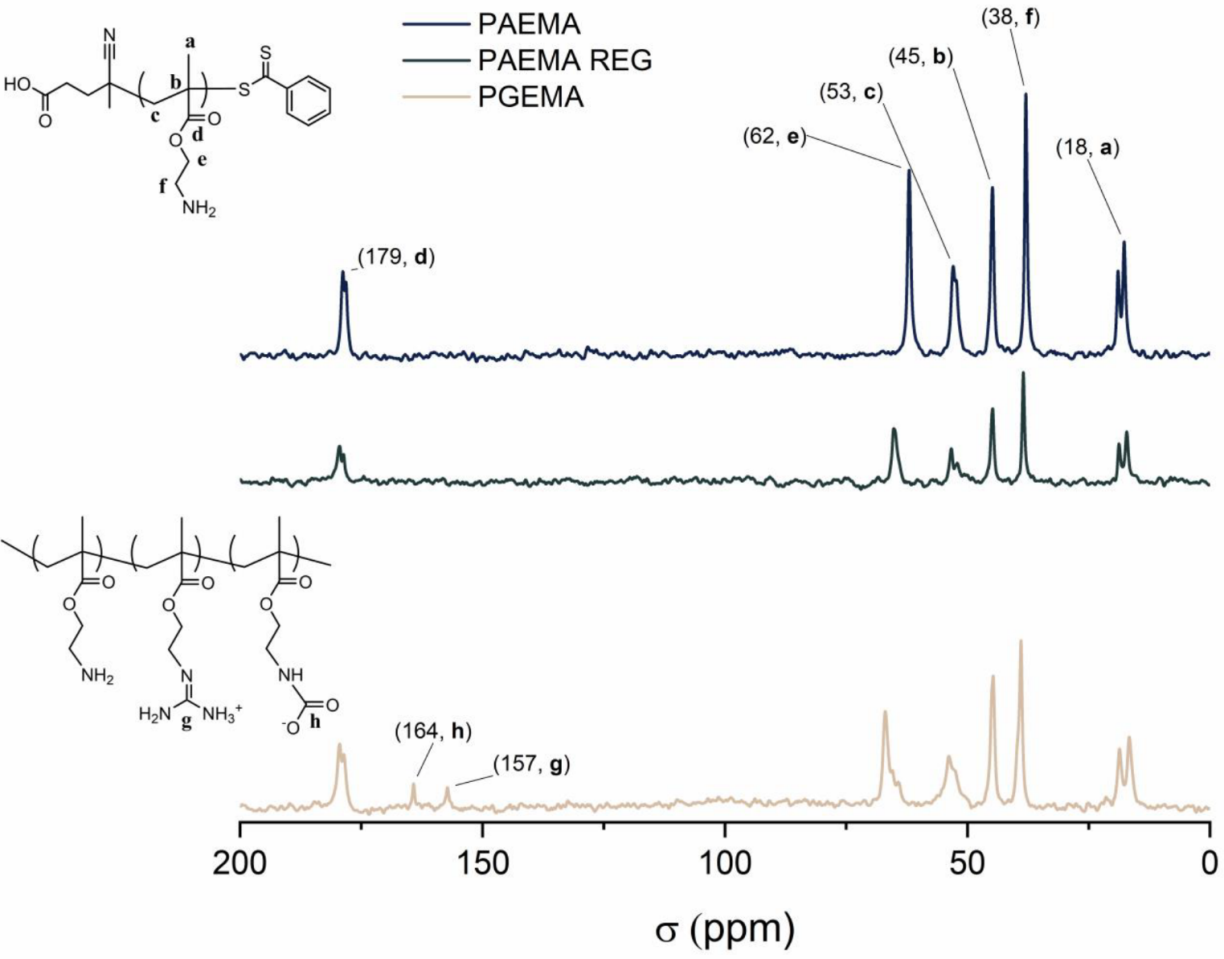
^13^C NMR spectra of PAEMA, PAEMA REG, and PGEMA.

The ^13^C NMR results of PGEMA show a signal at 157 ppm corresponding to the guanidine central carbon (—N=***C***—(NH_2_)(NH_3_)^+^) (Figure 3). Also, carbamate signals (CH_2_—NH—***C***OO^–^) at 164 ppm can be seen in PGEMA. The carbamate formation during guanidinylation is caused by the bicarbonate in the buffer regenerating the amine groups, producing water and CO_2_, which can then form carbamates with available amines.^41^ For the block copolymers PEO–PAEMA, PEO–PAEMA REG, and PEO–PGEMA, the NMR results are similar to the exception of small shift differences and a strong signal from the PEO chain (—***C**H*_2_—) at 3.65 ppm in the proton and 69 ppm in the carbon spectra (Figures S6 and S7).

**Figure 4:**
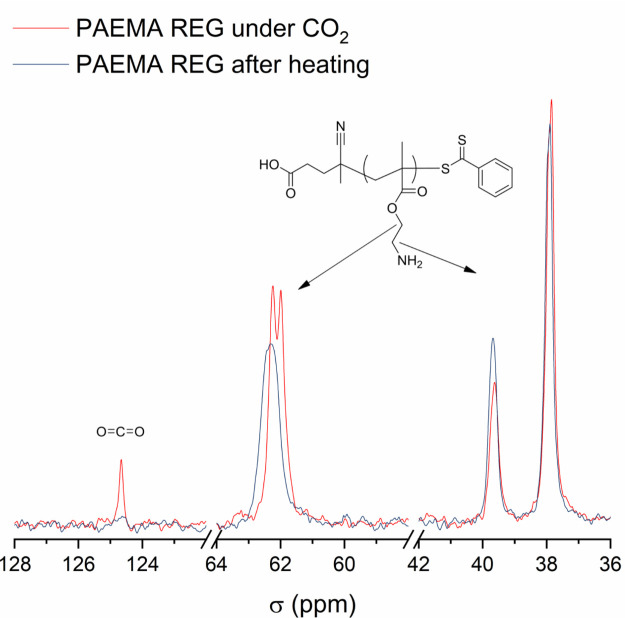
^13^C NMR of PAEMA REG under CO_2_ and after heating.

**Figure 5:**
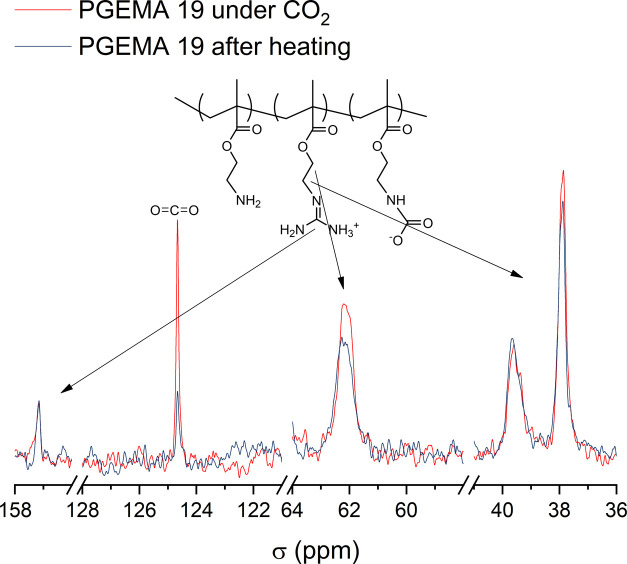
^13^C NMR of PGEMA 19 under CO_2_ and after heating.

To better understand the interactions of the polymers with CO_2_, ^1^H and ^13^C NMR measurements in air and under CO_2_ were conducted for PAEMA REG and PGEMA 19. When comparing samples subjected to CO_2_ and samples after heating at 60 °C in D_2_O solution and open air for 1 h to remove CO_2_, their ^1^H signals do not change between the two measurements, but differences are found in ^13^C NMR. Small changes in ^13^C signal shapes and shifts of groups located near amine/guanidine are observed (Figures 4 and 5).

The CO_2_ signal can be seen in PGEMA 19 and PAEMA REG at 124.5 ppm (Figures 4 and 5) before heating. This signal disappears from PAEMA REG when heated (Figure 4) but only dampens in PGEMA 19 (Figure 5). Clearly, PGEMA has a higher affinity to CO_2_. The groups near the amine are affected more by CO_2_ than those in the vicinity of guanidine. In PAEMA, CO_2_ changes the chemical shifts and shapes of the signals at 38 and 62 ppm, which correspond to the methylene carbons next to the amine (Figure 4). The same signals do not change as significantly in the case of PGEMA, which indicates a different stability of interaction (Figure 5). Consequently, in PGEMA, the signal corresponding to the guanidine at 157 ppm has a shoulder when under CO_2_ suggesting an interaction (Figure 5). Steric hindrance plays a part in the adsorption of CO_2_ in polymers.^41,60,61^ These results show that when the bulky guanidine is present, CO_2_ does not considerably change the environment of the groups close to the main chain. Also, although the nonguanidinylated side groups in PGEMA are primary amines, we do not observe similar changes in the signals at 37 and 62 ppm in PAEMA REG and PGEMA.

This suggests that the guanidine alters the interactions of CO_2_ with the amines. In conclusion, the differences in the chemical shifts and shapes in ^13^C NMR under CO_2_ and air give light to the interactions of the basic side groups with the acidic CO_2_. They also reveal that, on one hand, guanidine induces stronger CO_2_ binding, and on the other hand, simultaneously due to carbamate formation, some of the free amine functionalities are bound to a relatively stable carbamate, preventing them from further interacting with CO_2_.

### Gas Adsorption Studies

Prior to the gas adsorption studies, the thermal stabilities of PAEMA and PEO–PAEMA were measured by TG to rule out the polymer degradation affecting the results. The thermogravimetric runs of the polymers reveal a two-step degradation pattern consisting of the polymer side group cleavage beginning at 210 °C and the degradation of the polymer main chains at 320 °C (Figure S9),^62^ well above the temperatures used in the adsorption studies. At the beginning of the runs, the evaporation of water and adsorbed air can be observed, which was then accounted for by taking preparative heating and vacumization steps.

**Scheme 4:**
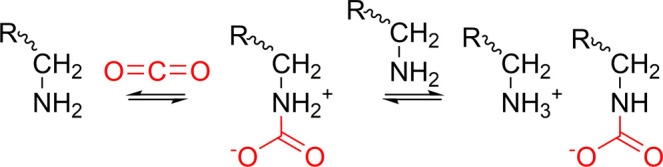
CO_2_ Adsorption with Primary Amines in Anhydrous Conditions

**Figure 6:**
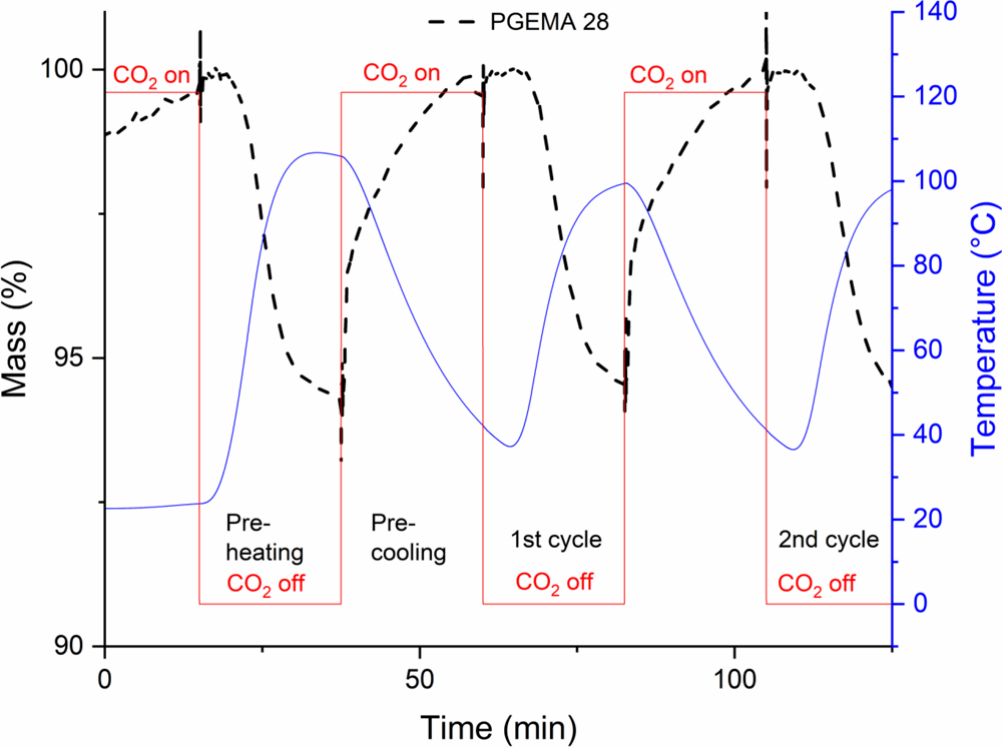
TG gas adsorption–desorption sequence and the mass changes of PGEMA 28.

**Figure 7:**
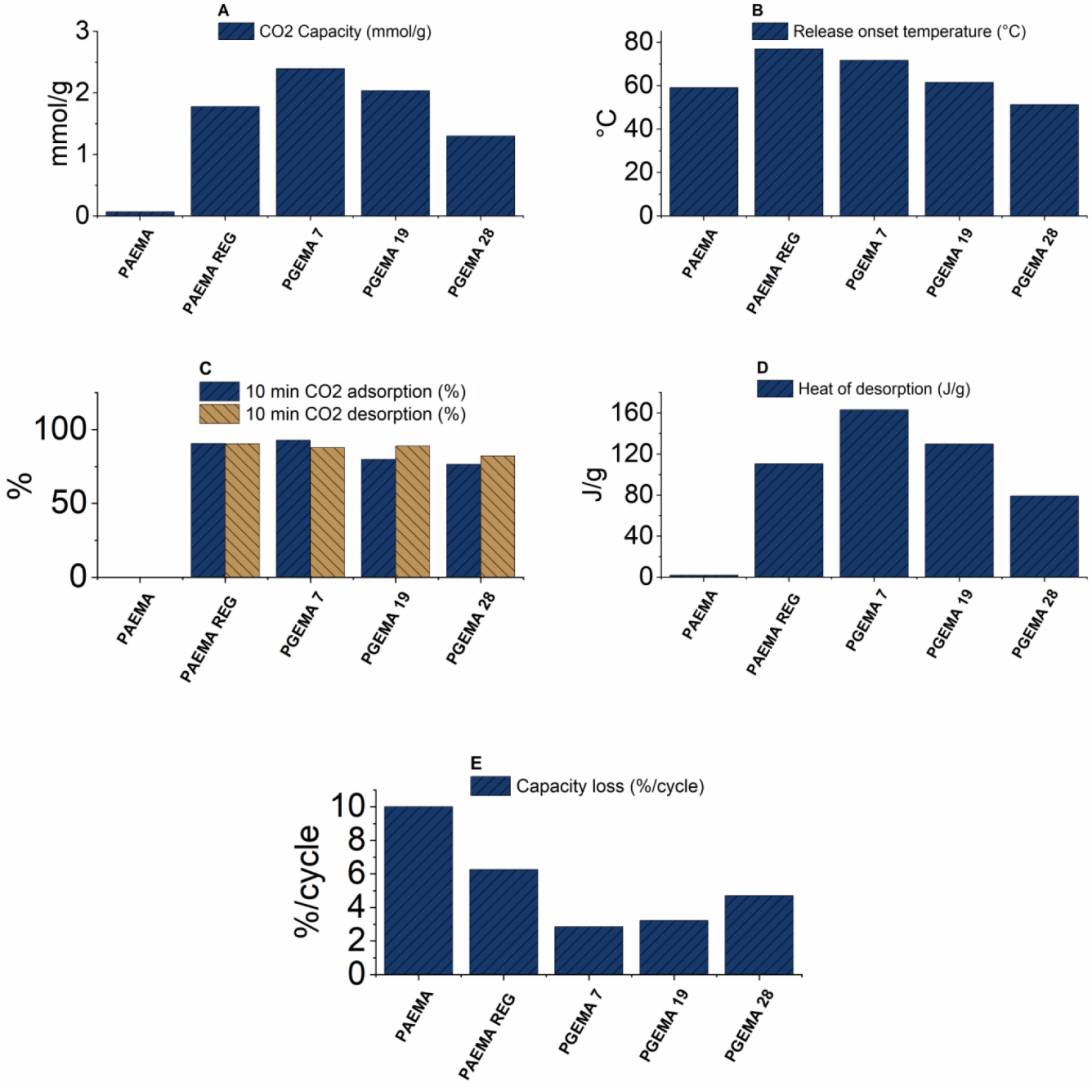
TG data of PAEMA homopolymers visualized: (A) CO_2_ capacity, (B) desorption temperature, (C) 10 min capacity, (D) heat of desorption, and (E) recyclability during one cycle.

**Figure 8:**
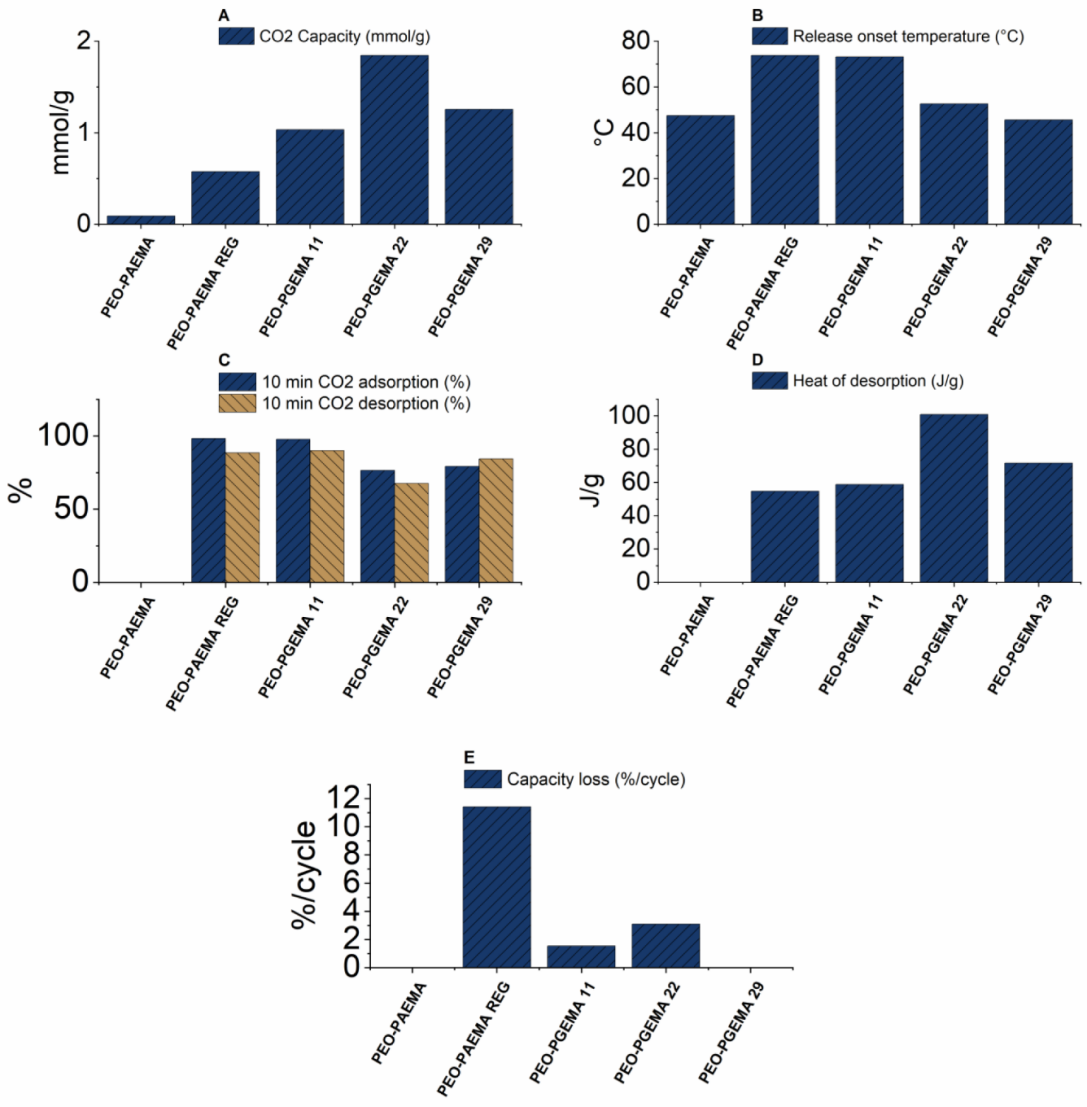
TG data of the PEO–PAEMA copolymers visualized: (A) CO_2_ capacity, (B) desorption temperature, (C) 10 min capacity, (D) heat of desorption, and (E) recyclability during one cycle.

The adsorption data for the studied polymers are shown in Figures 6–8 and Table 3. N_2_ adsorption measurements revealed minimal adsorption in all polymer samples (Figure S10), which is promising considering possible gas separation applications. However, with CO_2_, there were significant differences between the samples. The adsorption of CO_2_ to the salt form PAEMA and PEO–PAEMA was minimal, and most of the adsorption is most likely physical adsorption into the polymer matrix (Figures 7A and 8A). This is supported by the relatively low release temperatures of CO_2_ below 60 °C for both polymers (Figures 7B and 8B). When the polymer salts were regenerated into their free base forms, PAEMA REG and PEO–PAEMA REG, an impressive increase in CO_2_ capacity was observed. PAEMA REG has a capacity of over 1.7 mmol/g (increase of 200%) and PEO–PAEMA REG over 0.5 mmol/g (increase of 50%). These can be attributed to the increased amount of available amine groups for CO_2_ binding.^20^ According to Lee et al. and Chang et al., CO_2_ adsorption onto primary amines in anhydrous conditions forms a carbamate (Scheme 4).^25,30^

**Table 3:** Data Extracted from TG Measurements

sample	10 min absorption (%)	10 min release (%)	capacity loss (%/cycle)	release onset (°C)	heat of desorption (J/g)	capacity (mmol/g)	amine efficiency^a^ (CO_2_/NH_2_)
PAEMA	0^b^	0^b^	0^b^	59	2.0	0.07	0.01
PAEMA REG	90.5	90.4	6.3	77	110	1.8	0.29
PGEMA 7	92.7	87.9	2.8	72	163	2.4	0.40
PGEMA 19	79.7	88.8	3.2	62	129	2.0	0.34
PGEMA 28	76.4	82.1	4.7	51	79	1.3	0.22
PEO–PAEMA	0^b^	0^b^	0^b^	48	0^b^	0.09	0.02
PEO–PAEMA REG	98.3	88.4	11.4	74	55	0.6	0.10
PEO–PGEMA 11	97.8	90.1	1.5	73	59	1.0	0.18
PEO–PGEMA 22	76.5	67.5	3.1	53	100	1.8	0.31
PEO–PGEMA 29	79.2	84.4	0^b^	46	72	1.3	0.21

a Calculation based on theoretical 100% NH_2_ content. The theoretical values agree with actual amine content based on titration (Table S6).

b Values are not exactly 0 but are very small and can be considered negligible.

The interactions between the polymers and CO_2_ are also suggested by the increase in heat of desorption in DSC measurements (Figures 7D and 8D). Namely, the heat of desorption of CO_2_ is 110 J/g for PAEMA REG and 54 J/g for PEO–PAEMA REG compared to 2 J/g for PAEMA and ∼0 J/g for PEO–PAEMA. The temperatures of the onset of desorption of CO_2_ from TG are also increased due to the stronger interaction between the gas and the material (Figures 7B and 8B). When modified, the more basic guanidine increases the capacity up to a maximum of 2.4 mmol/g with 7% modification, a 35% increase compared to PAEMA REG. The capacity of PEO–PAEMA goes up to 1.8 mmol/g with a slightly higher 22% modification degree, a 260% increase compared to the unmodified PEO–PAEMA REG. The theoretical maximum amine efficiency of dry adsorption CO_2_ for primary amines is 0.5 CO_2_/NH_2_.^30^ For the polymers in this study, the efficiencies are 0.29 for PAEMA REG and 0.10 for PEO–PAEMA REG with increasing efficiency when modified (Table 3). This suggests that not all functional groups participate in the adsorption process. The morphologies of materials also have an effect on the available sites for adsorption. According to SEM, homopolymers PAEMA, PAEMA REG, and PGEMA 7 formed microspheres of size 1–5 μm, while an irregular granular morphology was observed in PEO–PAEMA, PEO–PAEMA REG, and PEO–PGEMA 22 (Figure S17). The differences between the homopolymer and block copolymer could be related to the crystalline nature of the PEO-block (Figure S15) affecting the morphology of the copolymer samples and lowering the adsorption performance.^55^

NMR measurements showed that guanidine functions assist in CO_2_ binding more strongly to adjacent amines than to plain amines in an aqueous environment. In this sense, it was interesting to observe the effect of the number of guanidines on the adsorption capacities in dry conditions. When increasing the amount of modified side groups, above 7% in PGEMA and 22% in PEO–PGEMA, the total adsorption capacity decreases accompanied with a lowering of desorption onsets and the heats of desorption. According to NMR studies, residual CO_2_ is present in the PGEMA solutions after heating (Figures 4 and 5). Residual carbamate is present in PGEMA after synthesis (Figures 1–3), which binds active amines, thus decreasing potential adsorption locations. However, heating should decompose carbamate and other forms of bound CO_2_ from the material.^18,42,63^ These results suggest that during heating not all of the adsorbed CO_2_ is released from the guanidine polymers, manifested as a lower overall capacity when measured from mass changes. As more guanidine is present, more CO_2_ remains adsorbed in the polymer in the carbamate form, lowering the observed capacity in TG measurements further. Wang et al. observed a slight increase in carbamate concentration in the first 30 min when desorption was conducted via heating, suggesting that our desorption time of 15 min at 100 °C could be too short to completely regenerate our samples.^64^ However, other factors may explain the results as well. Steric restrictions may play a part due to the bulky guanidine preventing access to some side groups and affecting adsorption.^41,60,61^ Another aspect is that guanidinylation may cause morphological changes in the material, for example cross-linking, which has been shown to decrease adsorption via fewer available amines, simultaneously increasing the desorption performance due to increased physical adsorption and less stable carbamates.^60,65,66^ Also, guanidines have been shown to form stable carbamates with amines, having higher sorption at temperatures over 50 °C suggesting that, in our experiments, the adsorption/desorption conditions for guanidinylated polymers are not optimal.^45^ Nevertheless, all samples reached over 80% of their apparent adsorption capacity in under 10 min, indicating an overall fast CO_2_ scavenging potential (Figures 7C and 8C).

The adsorbance–desorbance recyclability was assessed by comparing the adsorbance between two heating cycles. The adsorbance of PEO–PGEMA 29 stayed on the same level between the cycles, while PEO–PAEMA REG lost 11% adsorbance capacity/cycle (Figures 7E and 8E). The result for PEO–PGEMA 29 is promising, while a loss of 11% capacity/cycle is naturally unacceptable. Recyclability improved overall in both PGEMA and PEO–PGEMA where guanidine was introduced. Regarding the energy consumption, desorption temperatures and heats of desorption increased with regeneration but then decreased with guanidinylation. The homopolymers had higher capacities than PEO-block copolymers, but introducing PEO results in lower desorption temperatures and heats.

In conclusion, these results reveal complex interactions between CO_2_ and the functional groups in the polymers. The PAEMA polymers in their salt form do not adsorb CO_2_ practically at all; therefore, converting the polymers to the free base form is a prerequisite for adsorption. The adsorption capacities further increase with guanidinylation, but the maximum adsorptions are found with samples having only a low guanidinylation degree. On the other hand, desorption temperatures and heats decreased with guanidinylation and with the introduction of PEO.

## Conclusions

Poly(aminoethyl methacrylate) (PAEMA) and poly((ethylene oxide)-*block*-(aminoethyl methacrylate)) (PEO–PAEMA) were synthesized via RAFT in aqueous buffer. The polymers in their salt form after synthesis were regenerated to their free base form by treatment with a base. Guanidinylation (PGEMA and PEO–PGEMA) of both salt and free base forms of the polymers was then performed. The free base forms reacted faster and led to a higher degree of guanidinylation. NMR studies of PAEMA and PGEMA under CO_2_ and after heating revealed a stronger and more localized affinity of guanidinylated polymers toward CO_2_.

The CO_2_ and N_2_ adsorption capacities of polymers with amine hydrochloride functions, and those after regeneration to the free base form and guanidinylation, were studied. None of the polymers absorb N_2_, but their CO_2_ adsorption capacity increases first after regeneration and second after the introduction of guanidine. The maximum adsorption capacity of 2.4 mmol/g was found for samples with a relatively low degree of guanidinylation of 7%. Polymers with PEO-block demonstrated a lower adsorption capacity with a maximum CO_2_ adsorption capacity of 1.8 mmol/g.

CO_2_ desorption temperatures were below 80 °C for all of the polymers. The desorption onset of PAEMA, 77 °C, was lowered to 51 °C with guanidinylation. With PEO–PGEMA, the desorption temperatures were even as low as 46 °C with a high degree of guanidinylation. Heats of desorption are in line with the desorption temperatures.

The rates of CO_2_ adsorption and desorption increased after regeneration in both homo- and copolymer. The regenerated polymers lost and regained 90% of their capacity during the adsorption and desorption cycles within 10 min. Guanidinylation slowed both adsorption and desorption, and in 10 min, the polymers reached 75–88% of their capacity.

While the PAEMA polymers lost over 10% of their capacity after each cycle, guanidinylation improved stability considerably with losses of only 0–4.7% per cycle.

Overall, these polymers demonstrate promising properties for CO_2_ adsorption technologies, such as repeatable, relatively high and fast CO_2_ adsorption capacity and low desorption temperature.

## Supporting Information Available

Synthesis and characterization of the materials, CO_2_ adsorption studies by TG and DSC, amine group determination by acid–base titration, amine efficiency calculations, and SEM images of the polymer powders (PDF)
