# Supplementary material for: CO2 Capture and Low-Temperature Release by Poly(aminoethyl methacrylate) and Derivatives
Source: Langmuir. 2021 Dec 9;38(17):5197–208. doi: 10.1021/acs.langmuir.1c02321 (PMC9069862; doi:10.1021/acs.langmuir.1c02321)
Supplement: Supplementary file 1 — la1c02321_si_001.pdf [file la1c02321_si_001.pdf]

Supporting Information

CO_2 Capture and Low Temperature Release by Poly(aminoethyl methacrylate) and Derivatives

Tony Tiainen[1], Jere K. Mannisto[1], Heikki Tenhu[1], Sami Hietala*[1]*

[1]University of Helsinki, Department of Chemistry, P.O. Box 55, FIN-00014 HU, FINLAND

** heikki.tenhu@helsinki.fi, sami.hietala@helsinki.fi*

1. Synthesis

2. Characterization

3. CO_2 TG

4. CO_2 DSC

5. Amine group determination by acid-base titration and amine efficiency calculations

6. SEM

1. Synthesis

Buffers:

**250 mL Acetate buffer 1 M, pH = 5**					
Chemical	*c (mol/L)*	*n (mol)*	*M (g/mol)*	m (g)	*V (L)*
CH_3COOH	0.26997	0.06749	60.05	4.053	
$NaCH_3COO$	0.73000	0.18250	82.128	14.99	
H_2O					0.25

To prepare the acetate buffer for the polymer synthesis, 4.053 g of acetic acid (0.135 mol, 0.27 mol/L) and 14.9884 g of sodium acetate (0.365 mol, 0.73 mol/L) were dissolved into UHQ H_2O. After equilibration, the volume was adjusted to 250 mL. The pH of the buffer was determined to be 5.008.

**250 mL Carbonate buffer 0.1 M, pH = 10.2**					
	c (mol/L)	*n (mol)*	*M (g/mol)*	m (g)	*V (L)*
$NaHCO_3$	0.04614	0.01153	84.01	0.979	
$NaCO_3$	0.05385	0.01346	105.99	1.42	
H_2O					0.25

To prepare the bicarbonate buffer, 0.969 g of sodium bicarbonate (0.012 mol, 0.0460 mol/L) and 1.427 g of sodium carbonate (0.0130 mol, 0.054 mol/L) were dissolved into UHQ H_2O. After equilibration, the volume was adjusted to 250 mL. The pH of the buffer was determined to be 10.185.

Table S1: Preparation of buffers.

Polymerization:

PAEMA						
Reagent	*n /*	*n (mmol)*	*M (g/mol)*	*m (g)*	*V(mL)*	*c (g/mL)*
AEMA in acetate buffer	600	30.1896	165.62	5.0000	50 mL	*0.1*
CPA	*1*	0.0503	279.38	0.0141		
VA-044	0.25	0.0126	323.33	0.0041		
Protective gas: Argon						
Reaction time : 24 h						
Reaction temperature 60 °C						

PEO-PAEMA						
Reagent	*n /*	*n (mmol)*	*M (g/mol)*	*m (g)*	*V(mL)*	*c (g/mL)*
AEMA in acetate buffer	600	30.1896	165.62	5.0000	50 mL	*0.1*
PEO-CTA	*1*	0.0503	6000	0.3019		
VA-044	0.25	0.0126	323.33	0.0041		
Protective gas: Argon						
Reaction time : 24 h						
Reaction temperature 60 °C						

Table S2: Recipes for polymerizations.

Regeneration:

Regeneration of free base from PAEMA/PEO-PAEMA REG						
Chemical	*n/*	*n (mol)*	*M (g/mol)*	m (g)	*V (mL)*	*c (mol/L)*
PAEMA/PEO-PAEMA	1	0.003019	165.62	0.500000		
NaOH	1	0.003019	40	0.120749	30.2	0.1
**Regeneration of free base from PGEMA/PEO-PGEMA REG**						
PAEMA/PEO-PAEMA	1	0.000584	171.2	0.100		
NaOH	1	0.000584	40	0.023	5.84	0.1
Reaction time: 2 h						
Regenerated polymers are denoted "REG"						

Table S3: Regeneration of functional groups.

Modification:

PGEMA 7, 19, 28[1]						
Reagent	*n /*	*n (mmol)*	*M (g/mol)*	*m (g)*	*V(mL)*	*c (mol/L)*
PAEMA (7,19)/PAEMA REG (28)	1	1.2076	165.62	0.2000	50 mL	
2-ethyl-2-thiopseudourea hydrobromide	*1*	1.2076	185.09	0.2235		
Bicarbonate buffer	1.24215	1.5000			15	0.1
Protective gas: -						
Reaction time : 6 h (7), 24 h (19), 25 h (28)						
Reaction temperature: RT						

PEO-PGEMA 11, 22, 29[1]						
Reagent	*n /*	*n (mmol)*	*M (g/mol)*	*m (g)*	*V(mL)*	*c (mol/L)*
PEO-PAEMA (11, 22)/PEO-PAEMA REG (29)	1	1.2076	165.62	0.2000	50 mL	
2-ethyl-2-thiopseudourea hydrobromide	*1*	1.2076	185.09	0.2235		
Bicarbonate buffer	1.24215	1.5000			15	0.1
Protective gas: -						
Reaction time : 6 h (11), 25 h (22, 29)						
Reaction temperature RT						

[1]Percentage of guanidinylated side groups from ^{13}C-NMR.

Table S4: Modification of polymers.

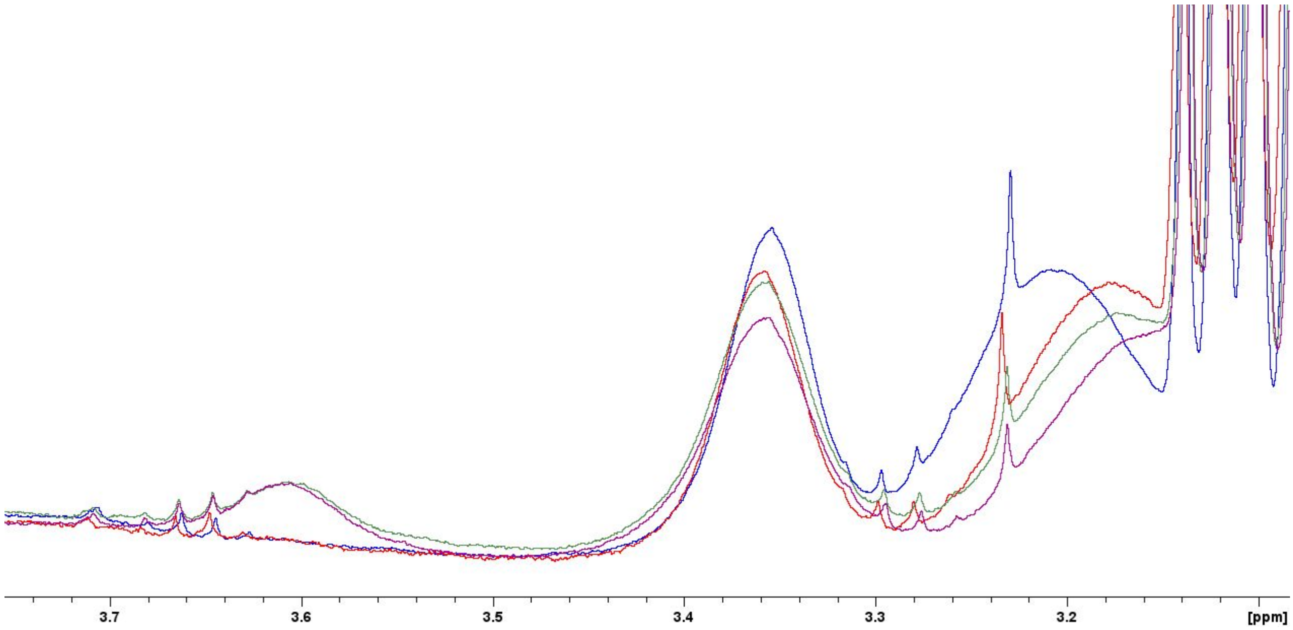

Figure S1: Overlay of samples taken during PGEMA synthesis at different intervals blue (15 min), red (1 h), green (20 h) and violet (24 h). Modification degree was calculated by comparing guanidine signal at 3.6 ppm to all side group CH_2 signals at 3.35- and 3.2 ppm.

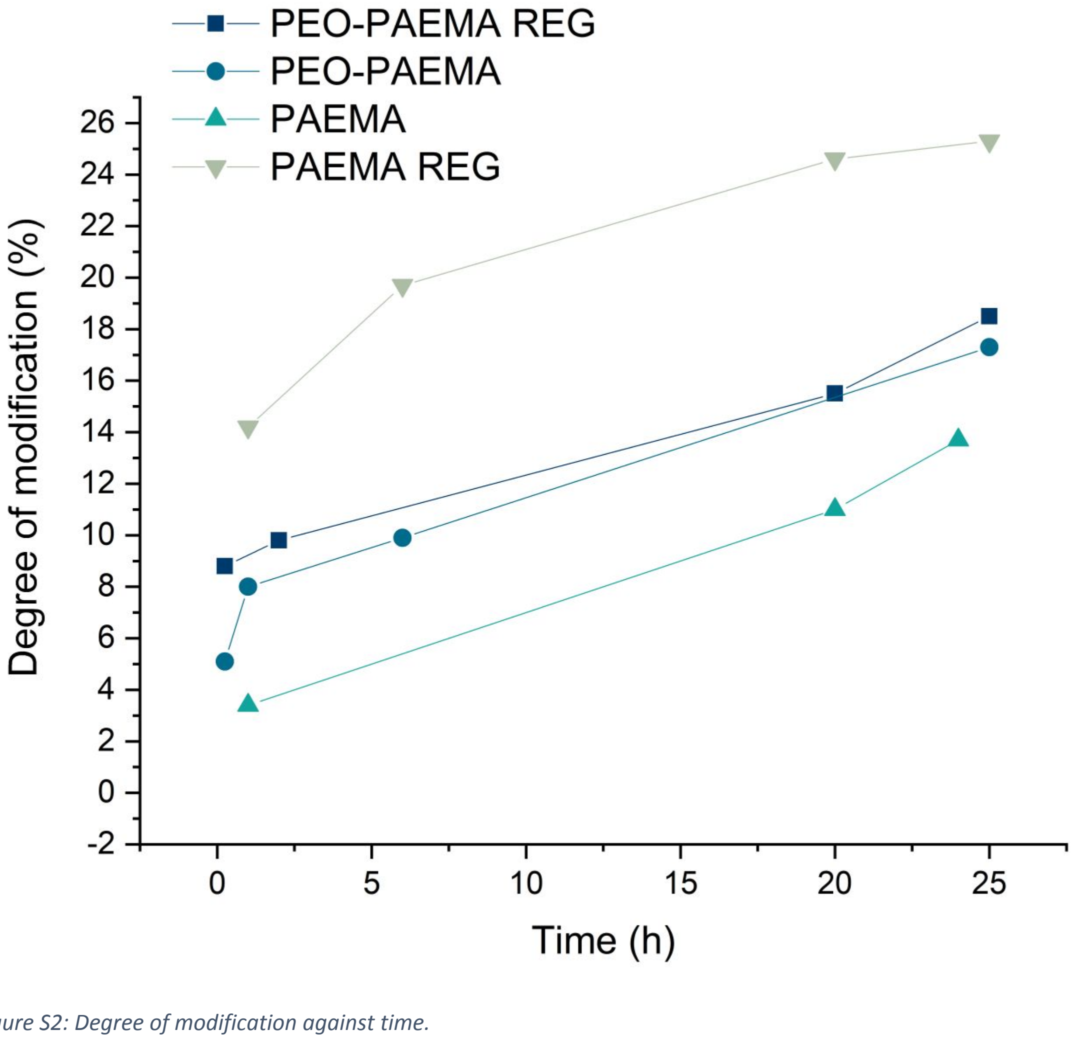

Figure S2: Degree of modification against time.

2. Characterization

AF4:

Flow program parameters				
Mode	*Time (min)*	*F_c (mL/min)*	*F_x (mL/min, start-end)*	F_i (mL/min)
Elution	5	0.5	5-5	0.2
Elution + Inject	15	0.5	5-0.2	0.2
Elution + Inject	5	0.5	0	0.2
Frit inlet channel				
dn/dc = 0.6841 g/mL				

Table S5: Flow program

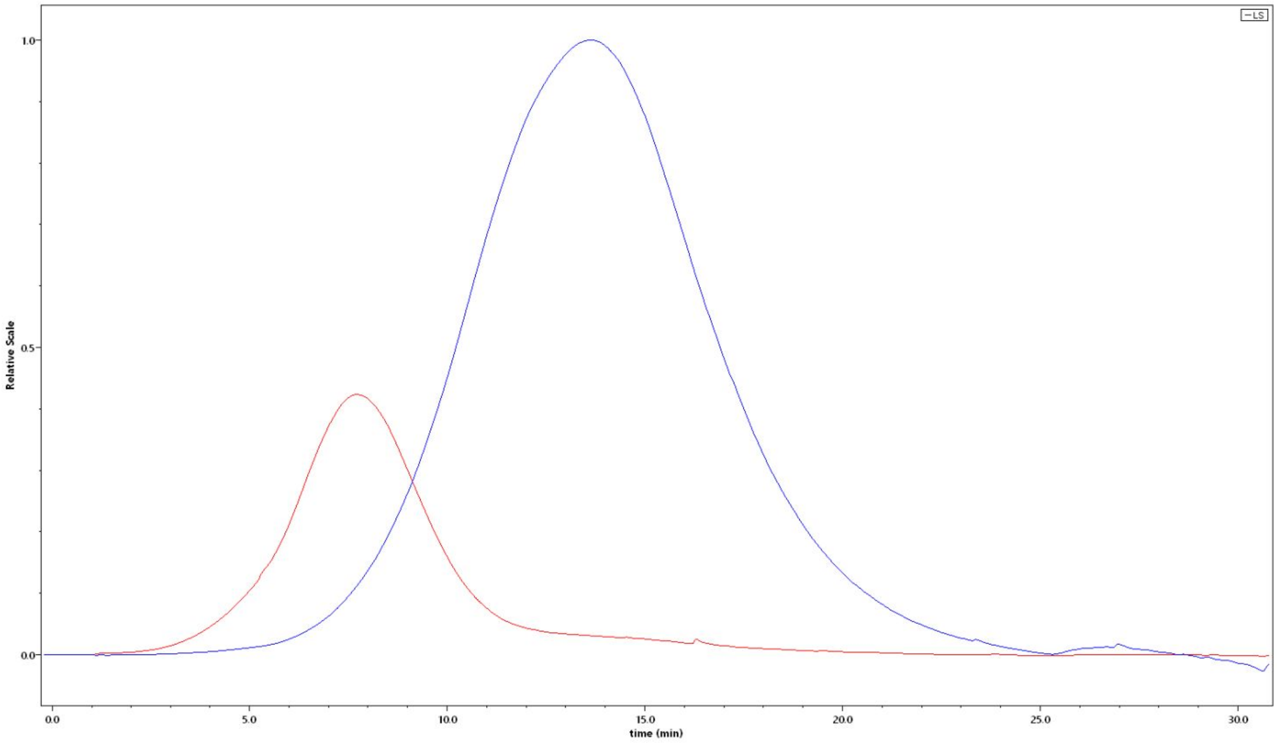

Figure S3: Fractograms of PAEMA (red) and PEO-PAEMA (blue).

IR:

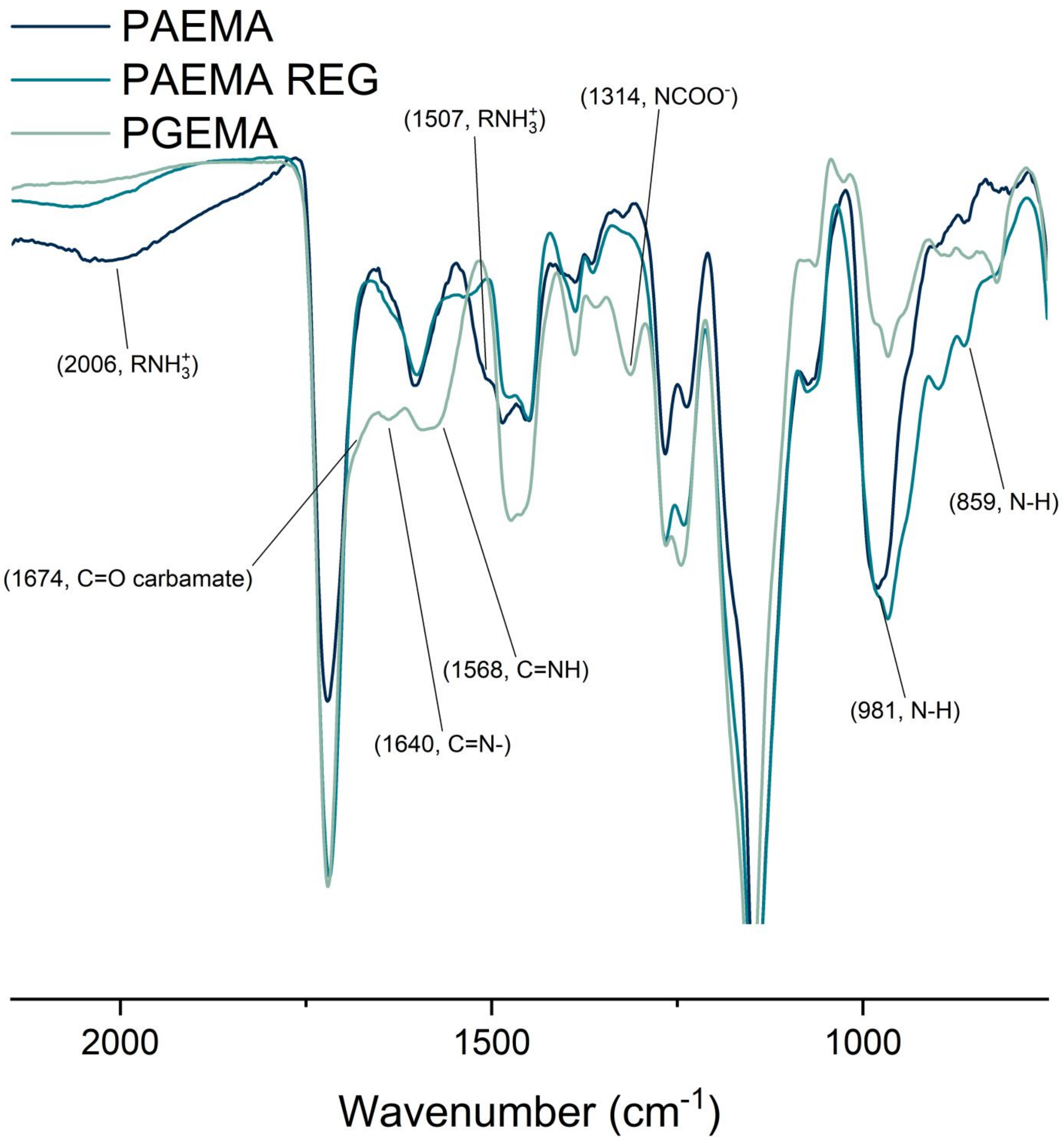

Figure S4: Overlay of IR-spectra from Figure 1.

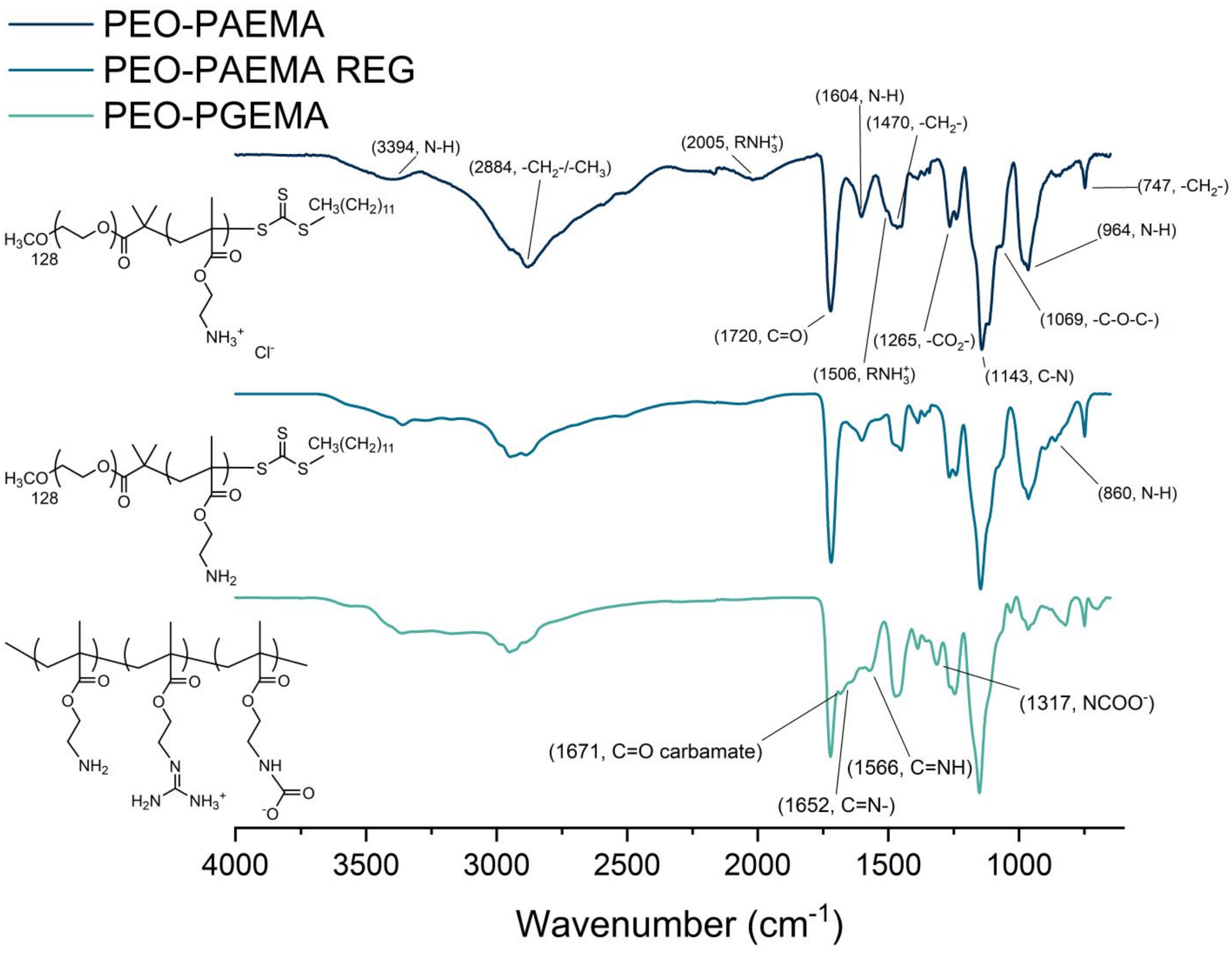

Figure S5: IR of PEO-PAEMA, PEO-PAEMA REG and PEO-PGEMA.

NMR:

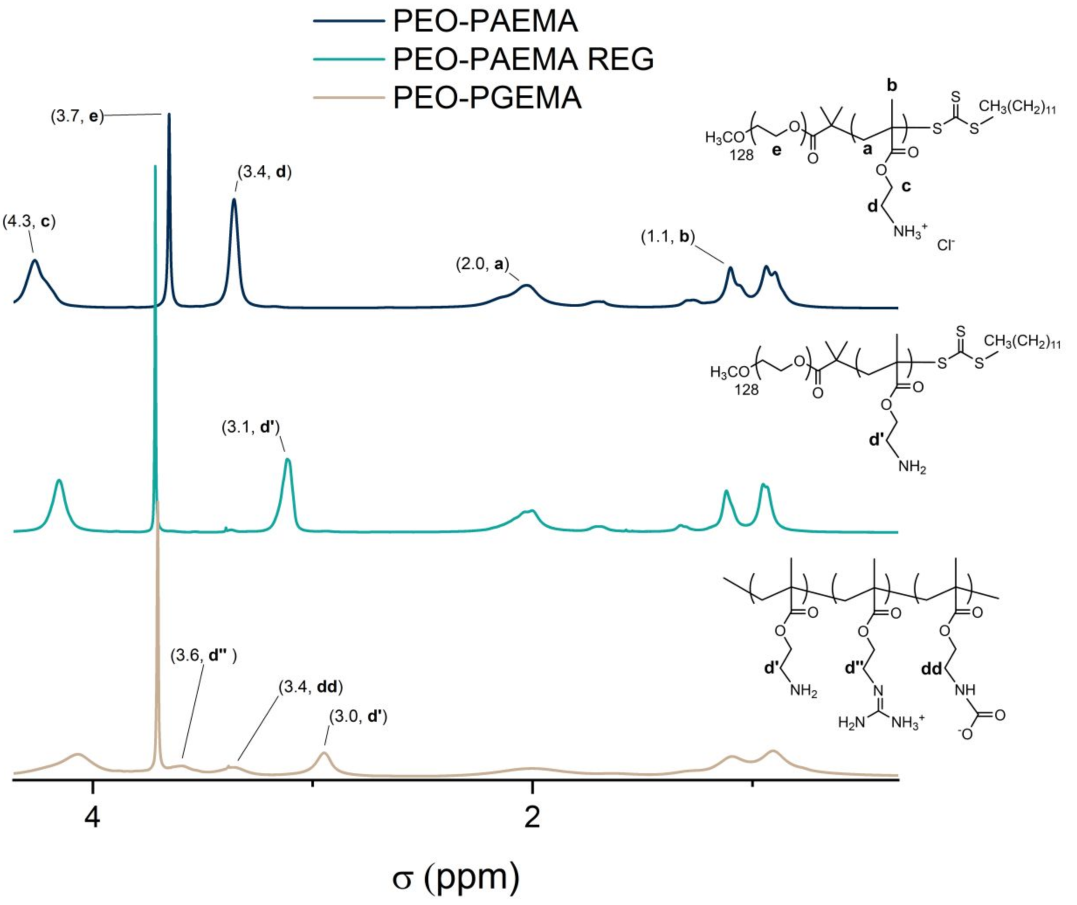

Figure S6: ^{1}H-NMR spectra of PEO-PAEMA, PEO- PAEMA REG and PEO-PGEMA.

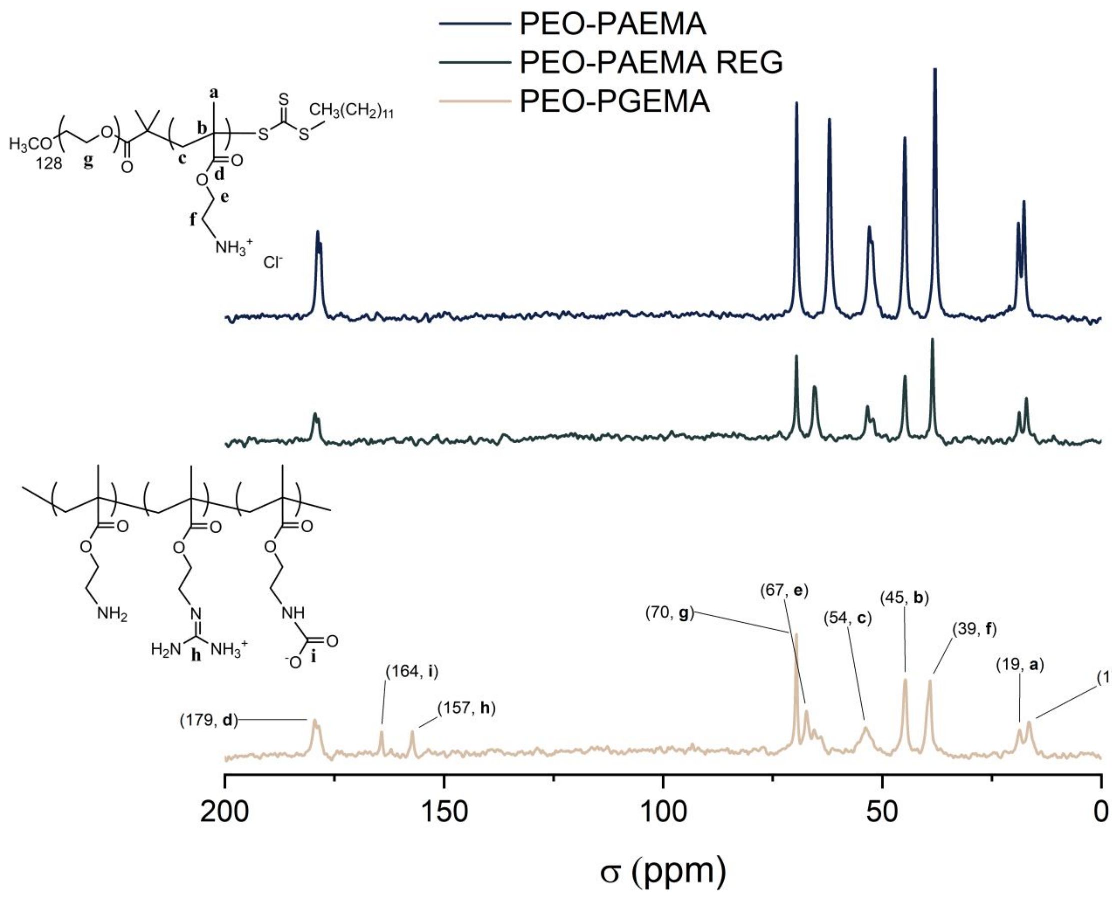

Figure S7: ^{13}C-NMR spectra of PEO-PAEMA, PEO- PAEMA REG and PEO-PGEMA.

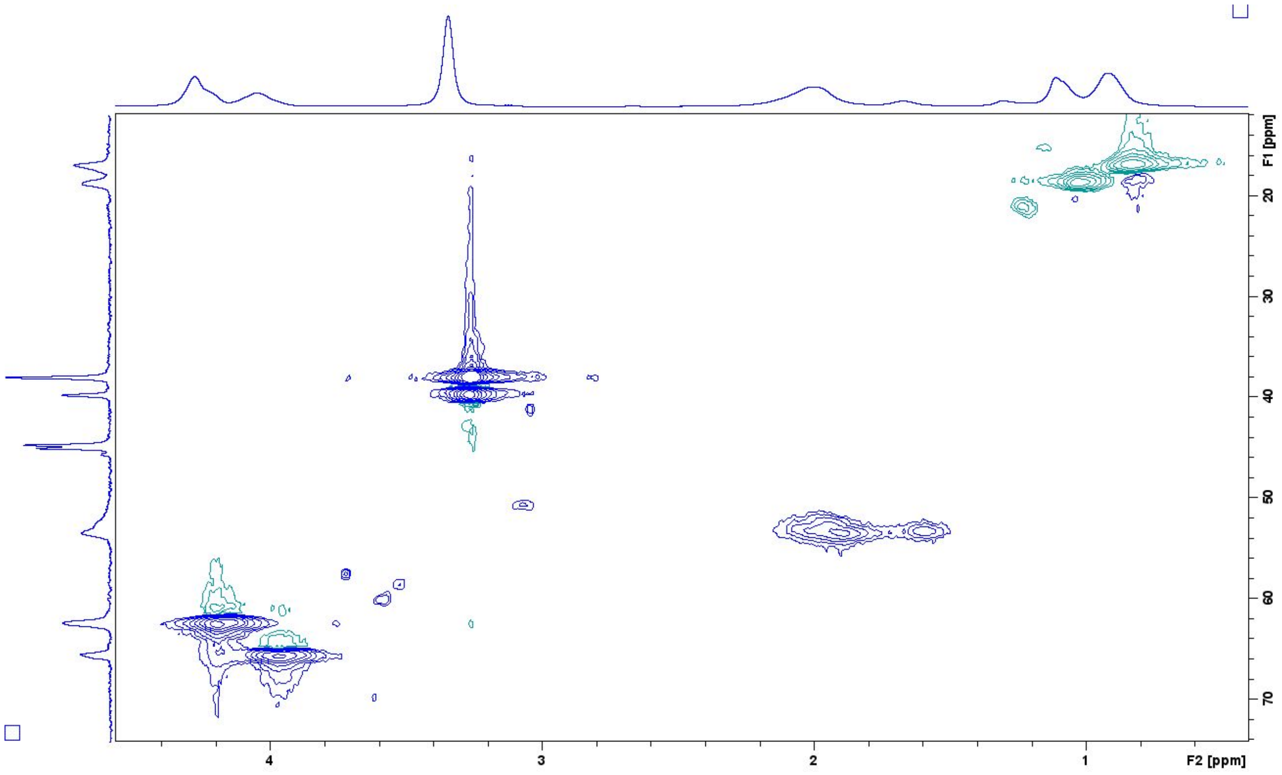

Figure S8: HSQC of PAEMA REG.

3. CO_2 TG:

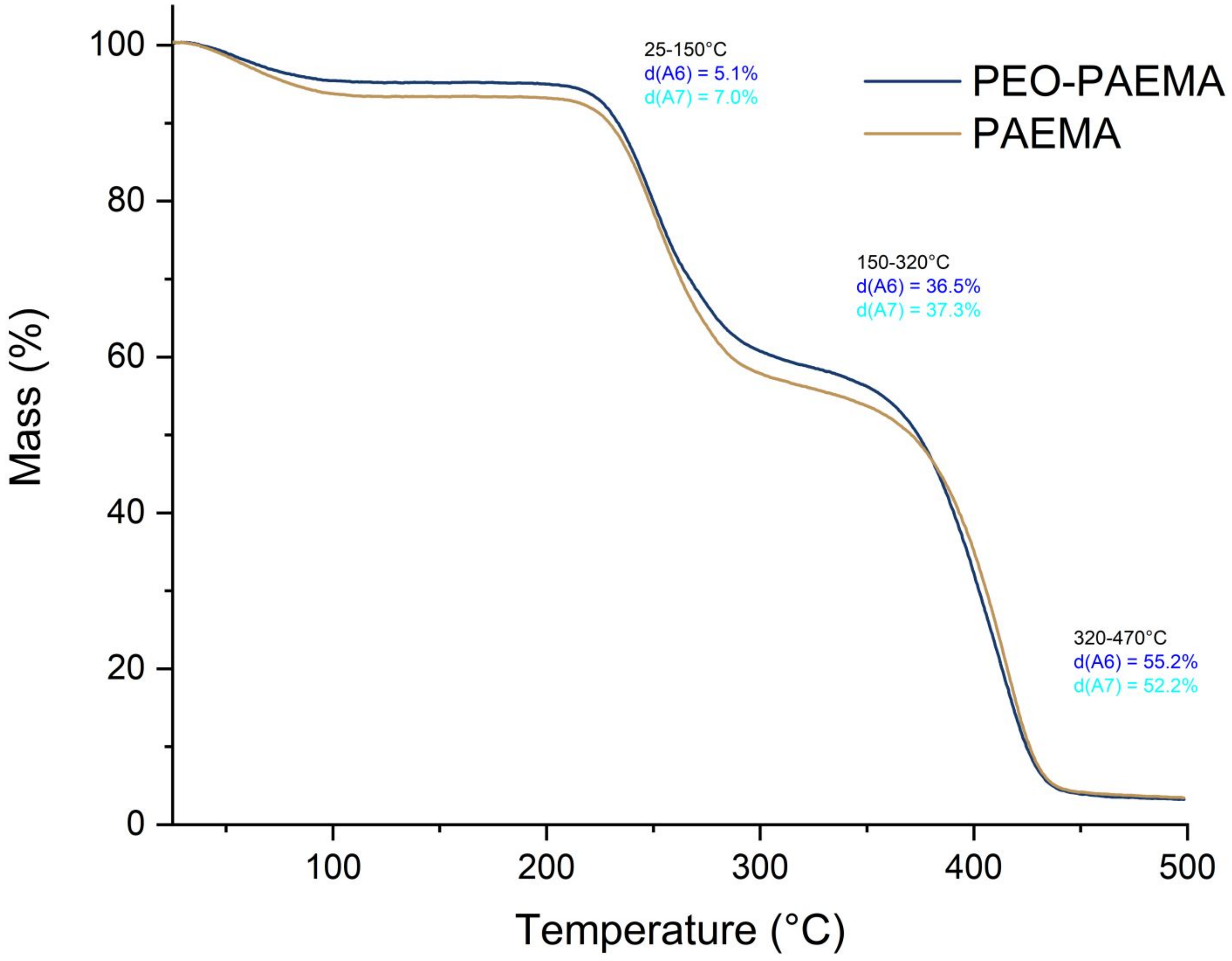

Figure S9: TG of PAEMA and PEO-PAEMA.

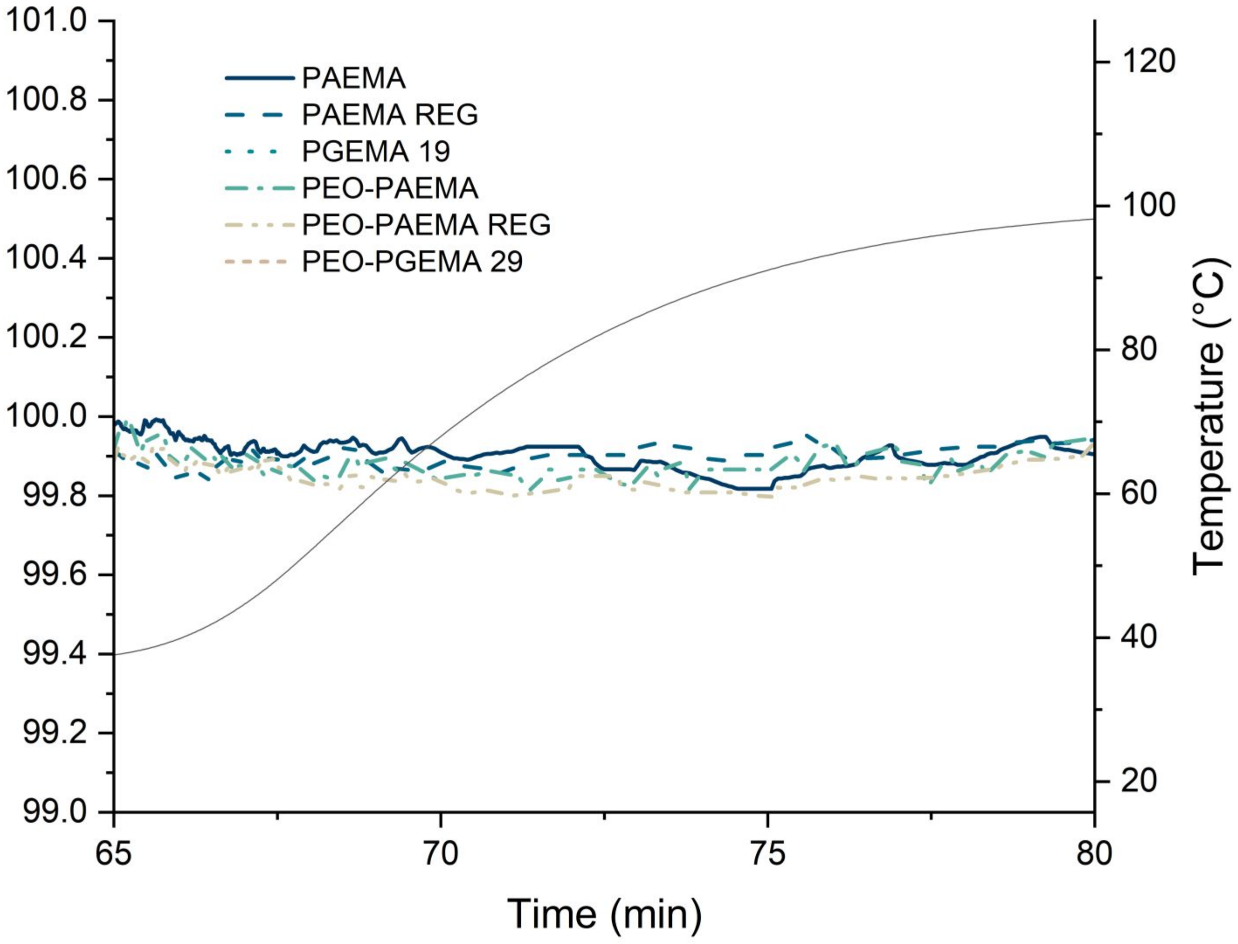

Figure S10: Samples heated after N_2 saturation.

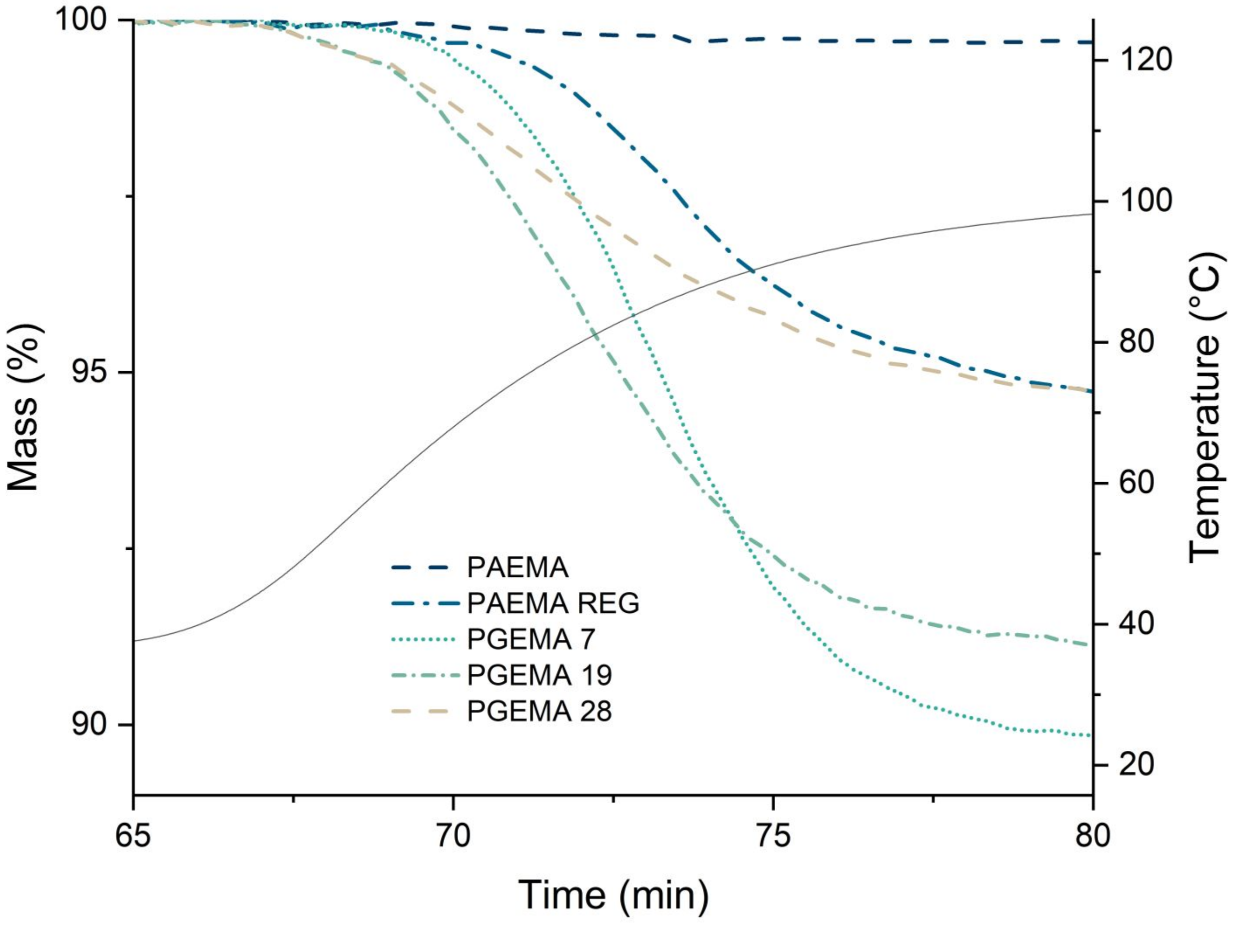

Figure S11: Desorption thermograms of homopolymers.

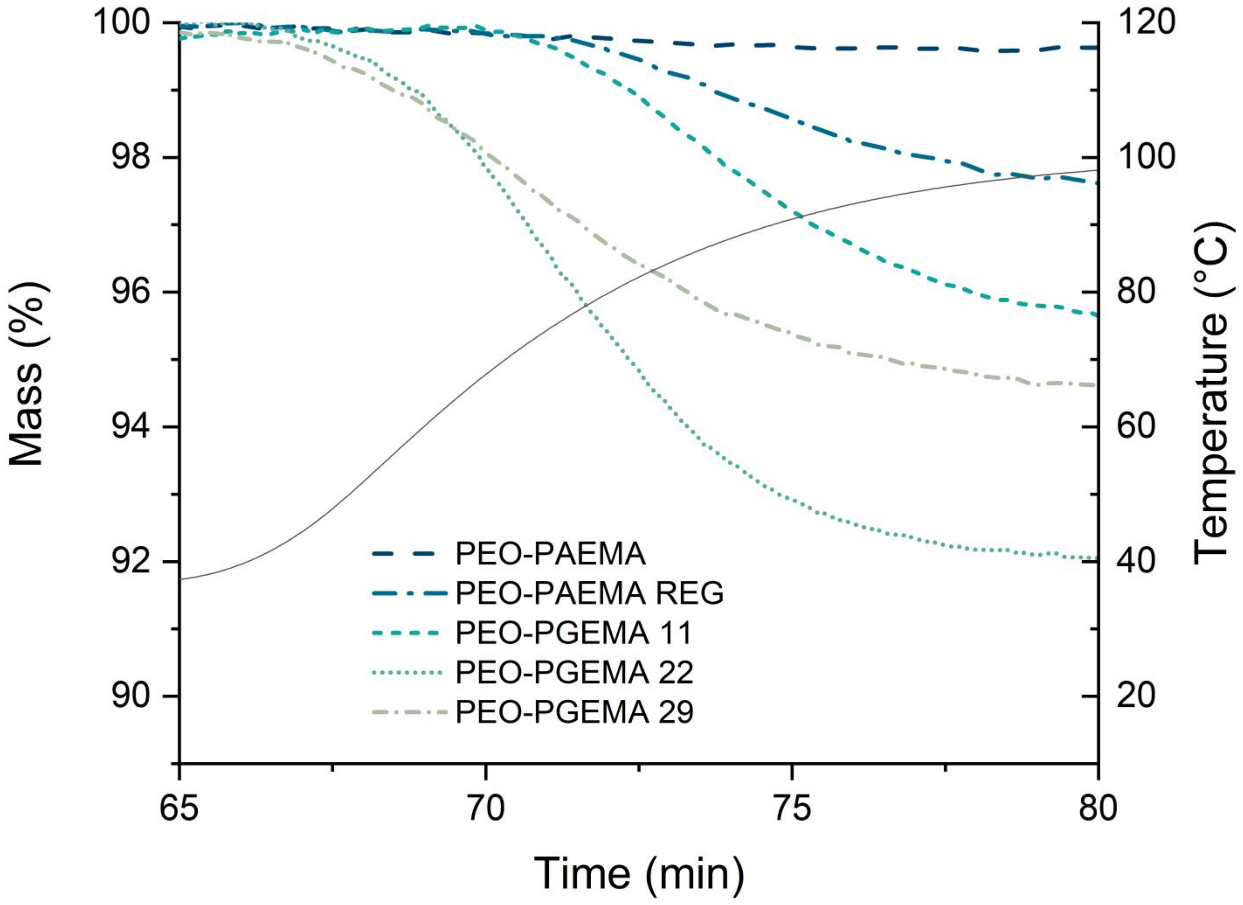

Figure S12: Desorption thermograms of the copolymers.

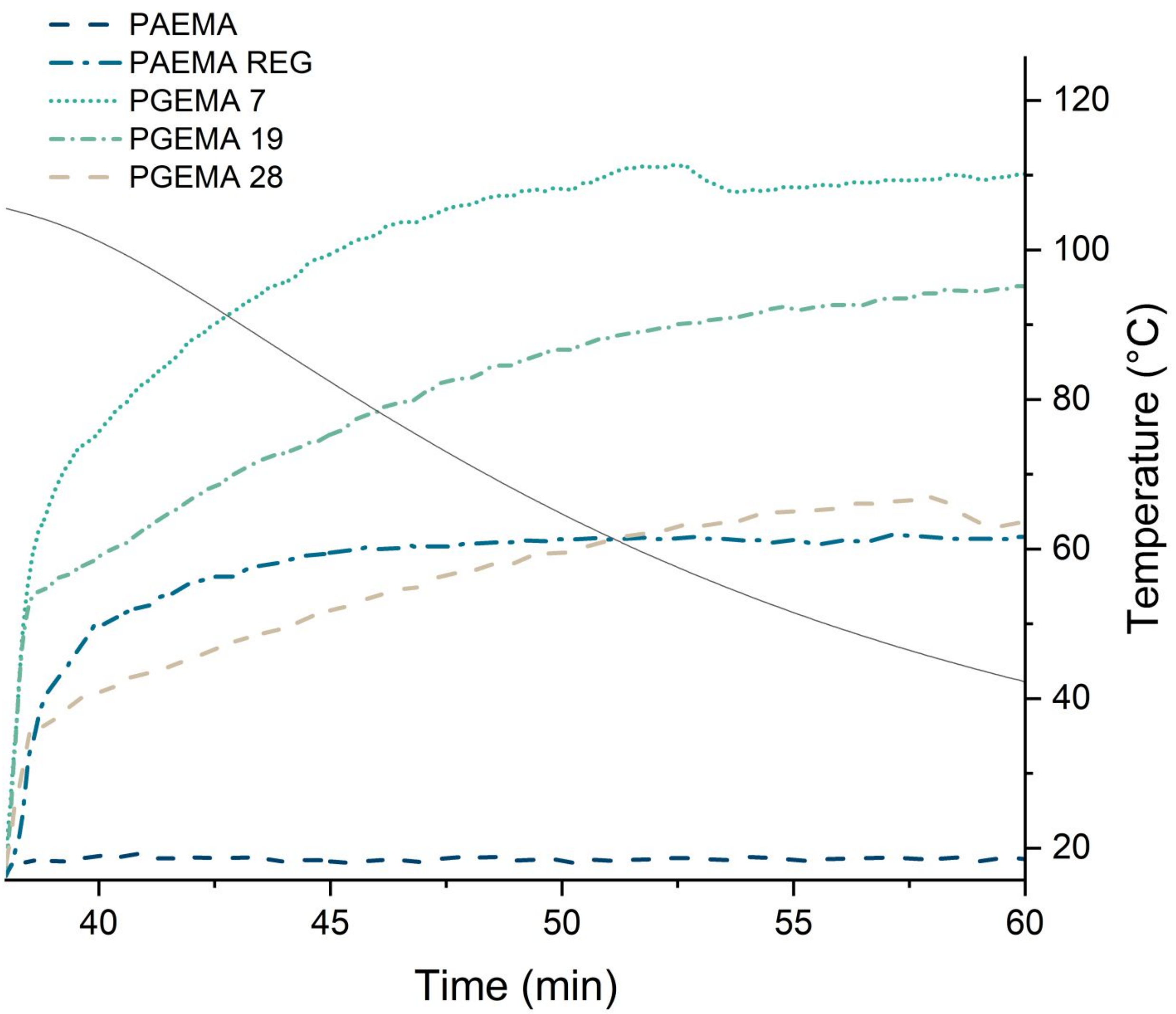

Figure S13: Adsorption thermograms of homopolymers.

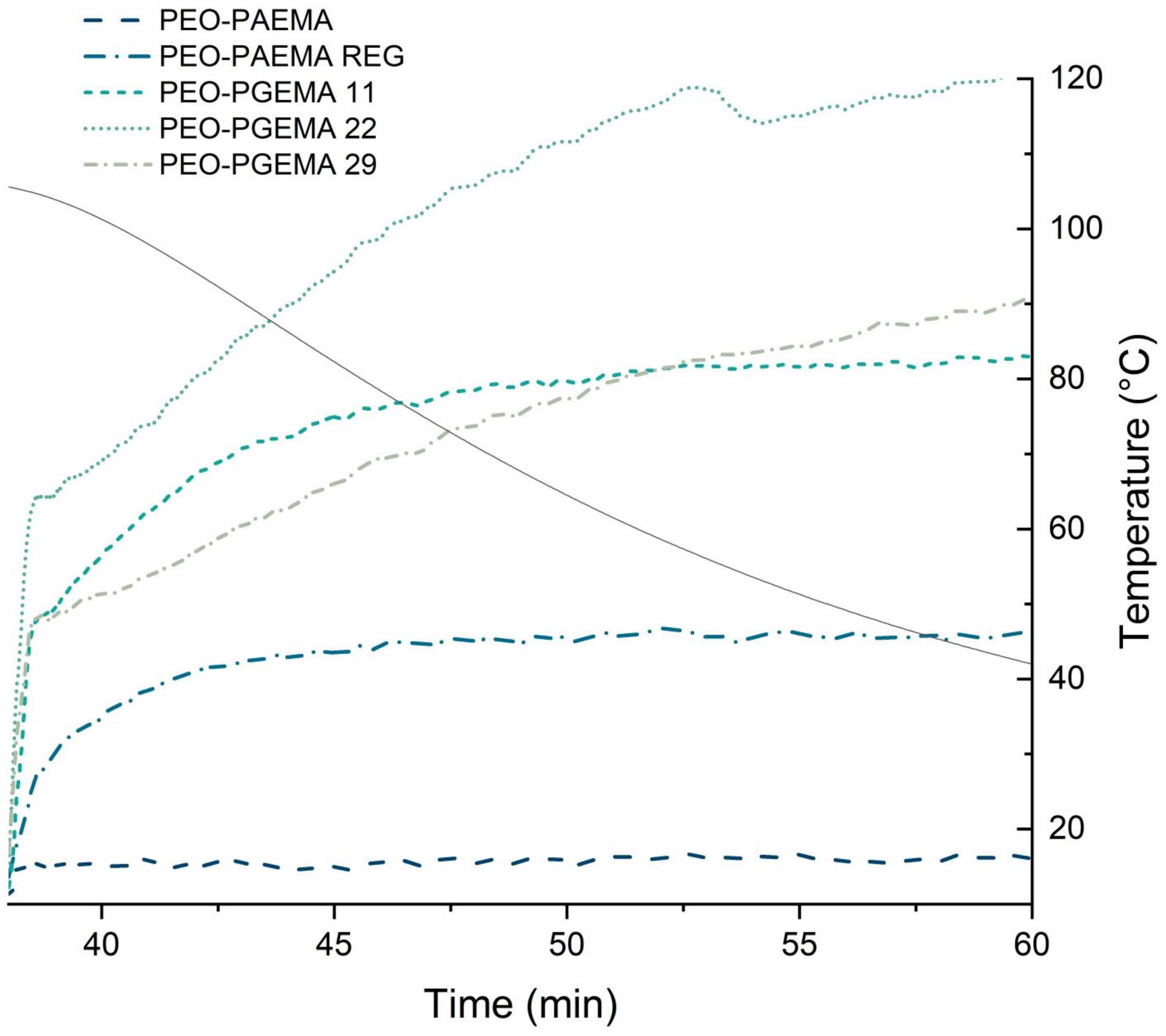

Figure S14: Adsorption thermograms of copolymers.

4. CO_2 DSC:

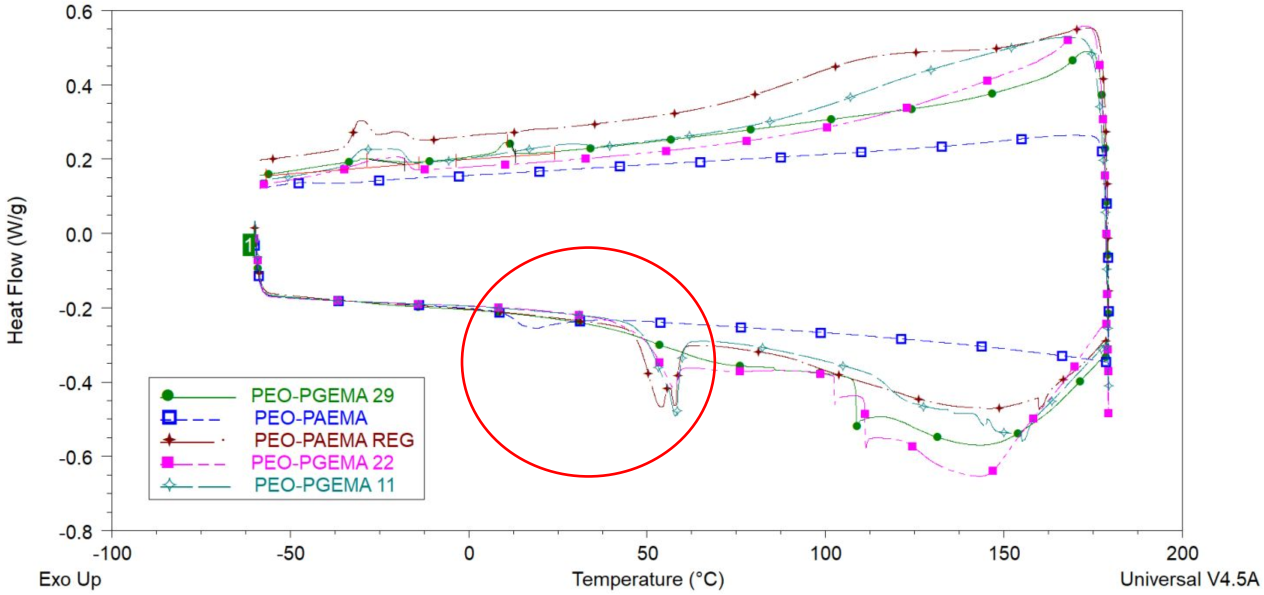

Figure S15: DSC-curves of the copolymers showing the melting and crystallization of PEO.

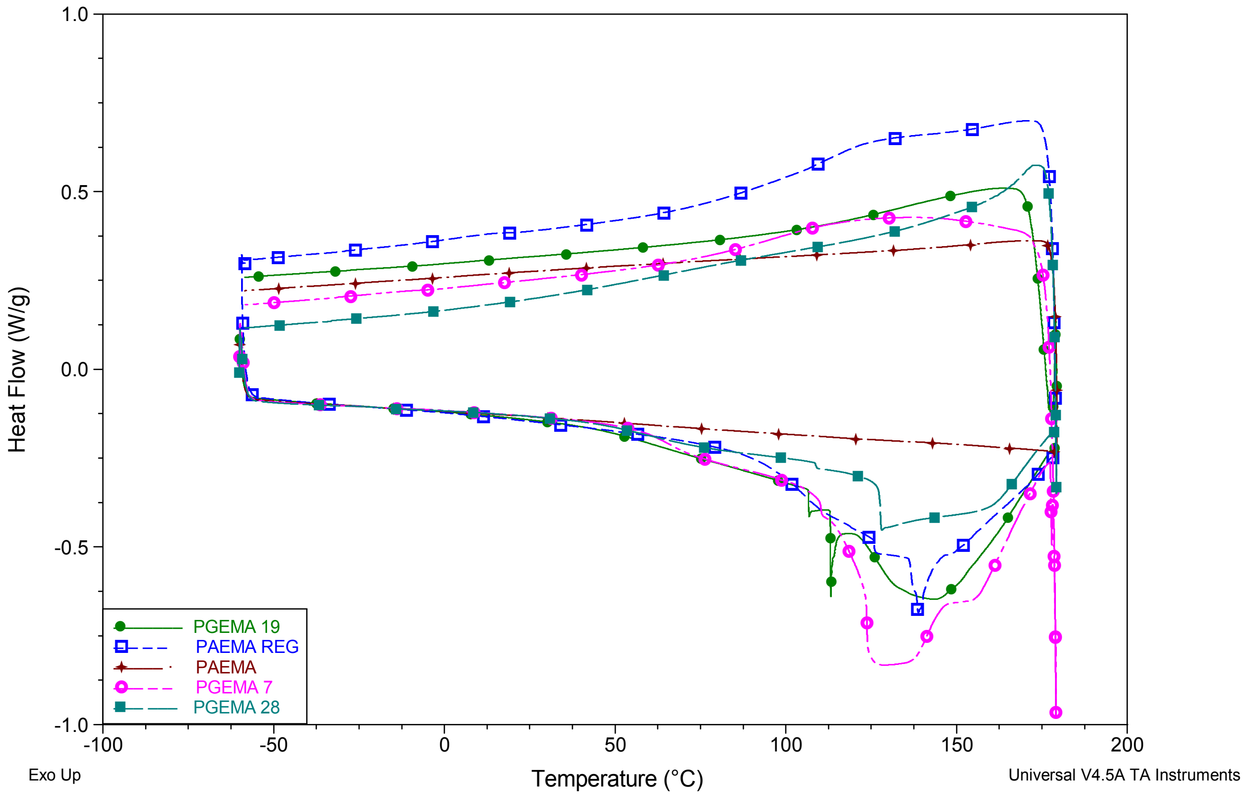

Figure S16: DSC-curves of homopolymers

5. Amine group determination by acid-base titration and amine efficiency calculations

Samples were dissolved in 0.01 M HCl by stirring for 2h. The acidic solution was titrated with 0.01 M NaOH and the volume of NaOH to reach the equivalence point was measured.

Amine group content

Sample	*Theoretical amount of amines in sample (mmol/g)*	*Amount of amines in sample (mmol/g)*	*Calculated amine efficiency (CO_2/NH_2)[1]*	*Amine efficiency (CO_2/NH_2)*
PAEMA REG	6	6.6	*0.29*	*0.27*
PEO-PAEMA REG	6	5.8	*0.10*	*0.10*

[1]Obtained from TG-data by calculating n(Amine)/n(CO_2) using the mass of sample and molar mass of repeating unit (165.62 g/mol) and mass and molar mass of CO_2 adsorbed.

Table S6: Acid-base titration of polymer samples.

6. SEM

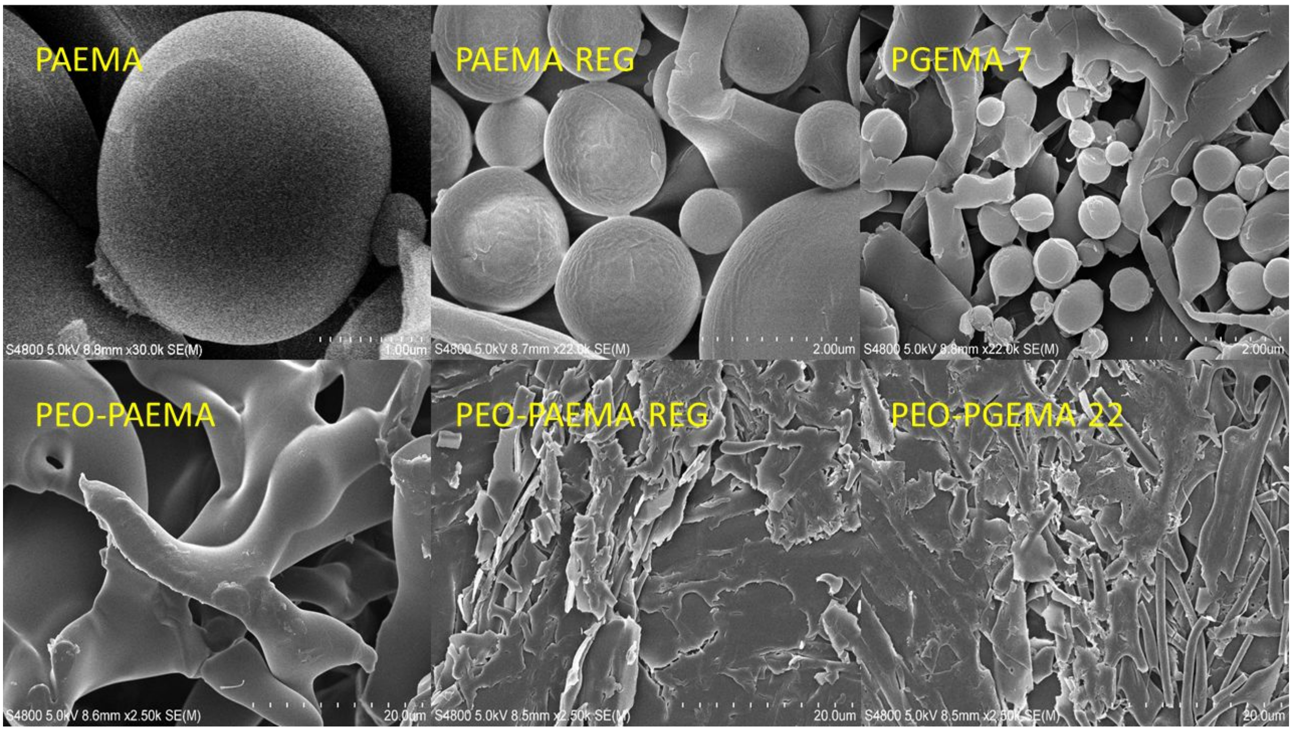

Figure S17: SEM images taken from sample powders. Homopolymers PAEMA, PAEMA REG and PGEMA 7 are microspheres and co-polymers PEO-PAEMA, PEO-PAEMA REG and PEO-PGEMA 22 are more irregular shape.
